# Glioblastoma cell motility depends on enhanced oxidative stress coupled with mobilization of a sulfurtransferase

**DOI:** 10.1038/s41419-022-05358-8

**Published:** 2022-10-30

**Authors:** Mirca S. Saurty-Seerunghen, Thomas Daubon, Léa Bellenger, Virgile Delaunay, Gloria Castro, Joris Guyon, Ahmed Rezk, Sylvie Fabrega, Ahmed Idbaih, Fabien Almairac, Fanny Burel-Vandenbos, Laurent Turchi, Eric Duplus, Thierry Virolle, Jean-Michel Peyrin, Christophe Antoniewski, Hervé Chneiweiss, Elias A. El-Habr, Marie-Pierre Junier

**Affiliations:** 1 CNRS UMR8246, Inserm U1130, Sorbonne Université, Neuroscience Paris Seine-IBPS Laboratory, Team Glial Plasticity and NeuroOncology, Paris, France; 2 CNRS UMR5095, Inserm U1029, Université de Bordeaux, Institut de Biochimie et Génétique Cellulaires, Team Bioenergetics and dynamics of mitochondria, Bordeaux, France; 3 ARTbio Bioinformatics Analysis Facility, Sorbonne Université, CNRS, Institut de Biologie Paris Seine, Paris, France; 4 Inserm U1312, Université de Bordeaux, Pessac, France; 5 Plateforme Vecteurs Viraux et Transfert de Gènes, Université Paris Descartes-Structure Fédérative de Recherche Necker, CNRS UMS3633, Inserm US24, Paris, France; 6 CNRS UMR 7225, Inserm U1127, Sorbonne Université, Institut du Cerveau et de la Moelle épinière, Paris, France; 7 Université Côte D’Azur, CNRS, INSERM, Institut de Biologie Valrose, Team INSERM Cancer Stem Cell Plasticity and Functional Intra-tumor Heterogeneity, Nice, France; 8 Service de Neurochirurgie, Hôpital Pasteur, CHU de Nice, Nice, 06107 France; 9 Service d’anatomopathologie, Hôpital Pasteur, CHU de Nice, Nice, 06107 France; 10 DRCI, CHU de Nice, Nice, 06107 France; 11 CNRS UMR8256, INSERM ERL1164, Sorbonne Université, Biological adaptation and aging-IBPS Laboratory, Team Integrated cellular aging and inflammation, Paris, France; 12 CNRS UMR8246, Inserm U1130, Sorbonne Université, Neuroscience Paris Seine-IBPS Laboratory, Team Axonal degeneration and regeneration, Paris, France

**Keywords:** CNS cancer, Cell migration, Cancer in the nervous system, Enzymes

## Abstract

Cell motility is critical for tumor malignancy. Metabolism being an obligatory step in shaping cell behavior, we looked for metabolic weaknesses shared by motile cells across the diverse genetic contexts of patients’ glioblastoma. Computational analyses of single-cell transcriptomes from thirty patients’ tumors isolated cells with high motile potential and highlighted their metabolic specificities. These cells were characterized by enhanced mitochondrial load and oxidative stress coupled with mobilization of the cysteine metabolism enzyme 3-Mercaptopyruvate sulfurtransferase (MPST). Functional assays with patients’ tumor-derived cells and -tissue organoids, and genetic and pharmacological manipulations confirmed that the cells depend on enhanced ROS production and MPST activity for their motility. MPST action involved protection of protein cysteine residues from damaging hyperoxidation. Its knockdown translated in reduced tumor burden, and a robust increase in mice survival. Starting from cell-by-cell analyses of the patients’ tumors, our work unravels metabolic dependencies of cell malignancy maintained across heterogeneous genomic landscapes.

## Introduction

Tumor development is a complex process into which the ability of cancer cells to adapt their behavior to the constantly changing environment of a growing tumor and to therapies plays a crucial role. Metabolism is central in shaping cell behavior. Therefore, its appropriate reprogramming is expected to be mandatory for cancer cell plasticity. Metabolism appears moreover as a target of choice to overcome the heterogeneous genomic landscapes prevailing in complex tumors. The mapping of metabolic reprogramming in cancers progressed remarkably in recent years [1–3**]**. However, the nature and the role of metabolic activities specific to a given cancer cell behavior at the single-cell level and in the actual context of the patients’ tumors remain poorly explored. This is notably the case regarding cancer cell motility, a functional state essential for tumor malignancy.

In this study we explored the metabolic dependencies of cancer cell motility in glioblastoma (GB). GB is the most aggressive tumor of the adult human brain [4] and is a paradigm of intra-tumor heterogeneity. It is composed notably of a mixture of cancer cells bearing various mutational loads and genomic rearrangements, and differing ontogenic similarities with neurodevelopmental lineages, as shown by genome-wide DNA and RNA sequencing at the tissue and single-cell levels [5–13]. GB is further characterized by the plasticity of its cancer cell populations that fuel tumor growth through reversible adaptations of their properties [9, 14, 15]. The highly infiltrative nature of GB cells makes complete surgical resection of the tumor impossible. Moreover, currently used irradiation and chemical therapies can promote GB cell invasiveness [16, 17]. Cell motility encompasses two processes: migration, i.e. the cell’s ability “to move around in a space that is freely available”, and invasion, a process involving microenvironment remodeling by the cells [18]. Cell motility is a highly integrated process, motile cells having to develop protrusions in the direction of migration, disrupt adhesion sites at the cell rear, degrade and remodel the extracellular matrix (ECM) and ultimately make new connections at the cell front by remodeling their cytoskeleton so that they are pushed forward [19]. Three major migration/invasion routes have been described in GB: the narrow and tortuous extracellular space of the brain parenchyma [20], the perivascular spaces surrounding blood vessels [20**–**23] and the white matter tracts [20, 24]. Both single-cell and collective migration have been reported [25, 26]. However, it is still poorly known whether cell motility in the patient’s tumor depends on specific metabolic reprogramming. We therefore chose to harness single-cell transcriptomes of GB cells derived from surgical resections of the patients’ tumors, in order to identify metabolic enzymes playing key roles in the adoption and/or maintenance of cell motility.

To unravel the metabolic dependencies of motile cells within the heterogeneous context of the patients’ tumors, we started from single-cell transcriptome profiling of thirty tumors coming from four independent datasets. A signature-driven data reduction-based analysis resulted in highlighting cell subpopulations most likely to be in a motile state. Computational analyses integrating cell trajectory modeling characterized motile cells as endowed with higher energetic needs and oxidative stress than their more static counterparts. This modeling further identified an element of the cysteine metabolism, the 3-Mercaptopyruvate sulfurtransferase (MPST) enzyme, at the crossroad of the path from low to high motility. Experimental probing of the biological relevance of these findings using a variety of functional assays resulted *in fine* in demonstrating MPST as a previously unsuspected key metabolic player in the switch from low to high cell motility and maintenance of GB cell motility.

## Results

### Grouping GB cells from distinct patients according to their motility potential

We first aimed at identifying cells in a motile state from single-cell transcriptomes coming from distinct patients’ tumors. For this purpose, we used four independent publicly-available single-cell transcriptome datasets generated from surgical resections of patients bearing IDH-wild-type GB. These datasets were obtained from 30 tumors with varying genomic and epigenetic anomalies, notably according to MGMT methylation, TP53 mutant or wild-type status, ATRX loss, Chr7 duplication or Chr10 deletion [9, 27] (Figs. S1A and S2A). Two datasets were obtained with the SMART-seq2 technology [9, 27] and two with the 10X Genomics technology [9, 28], hereafter designated as N-S, D-S, N-10X and PA-10X, respectively. The computational analyses were first performed using the N-S dataset, and the robustness of the results evaluated by repeating the analyses with each of the other three datasets.

**Fig. 1:**
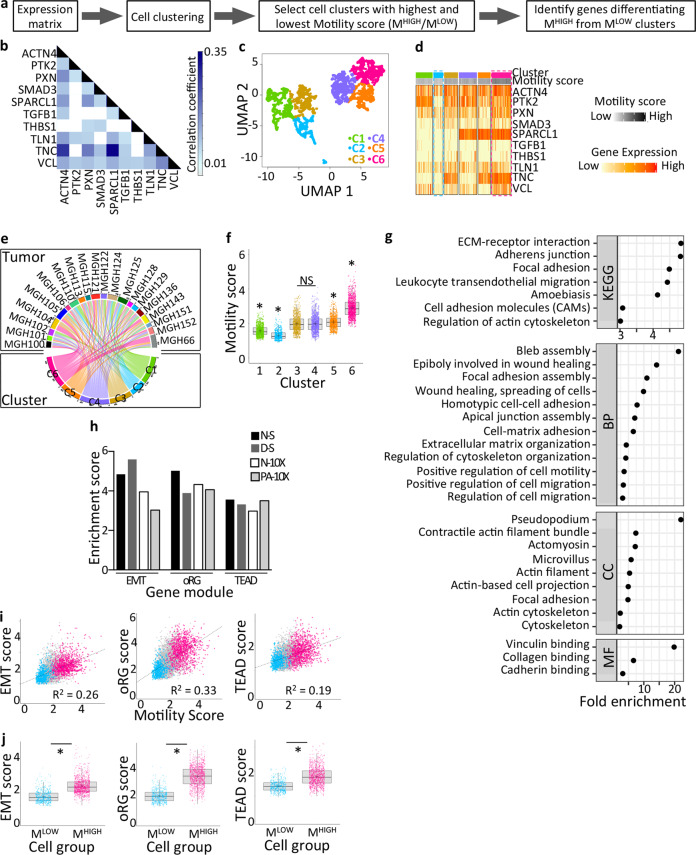
Grouping GB cells from distinct patients according to their motility potential. **a** Schematic outline of the computational analytical strategy. **b** Significant correlations between expressions of the ten genes of the motility signature (*p* < 0.01). **c** Malignant cell clustering based on the motility signature. **d** Different combinatorial expressions of the signature genes characterize each cluster. **e** Each cell cluster contains cells coming from distinct tumors (NMI Score=0.11). **f** Identification of cell groups with the highest and lowest mean motility scores (C6: M^HIGH^, C2: M^LOW^). *Clusters statistically different from each of the other clusters, *p*-value < 0.01, one-way ANOVA, Tukey’s multiple comparisons test. NS: non-significant. **g** Motility-related terms highlighted by ontology analysis of genes overexpressed in M^HIGH^ versus M^LOW^ cells. Genes overexpressed with fold change ≥2. BH-adjusted *p*-value < 0.05. BP biological processes, CC cellular components, MF molecular functions. **h** Enrichment in EMT, oRG, and TEAD gene modules previously associated with GB cell motility in M^HIGH^ cells. *p* < 0.001, hypergeometric test. **i** Linear regression model between motility score and EMT, oRG and TEAD scores. *p* < 0.0001. **j** Higher EMT, oRG and TEAD scores in M^HIGH^ versus M^LOW^ cells. *p < 0.0001, Mann–Whitney test. **b**–**g**, **i**, **j**: N-S dataset analysis. See also Figs. S1–5 and Tables S1–3.

For clustering cells in a motile state, we implemented a data reduction approach driven by a molecular signature (Fig. 1a). Cells were grouped based on the combined expression levels of each gene in a motility signature, using the HCPC algorithm (Hierarchical Clustering on Principal Components) modified to integrate also UMAP components (Uniform Manifold Approximation and Projection; Fig. S3A). The motility signature genes were selected based on prior in vitro and in vivo experimental demonstrations of their requirement for GB cell motility using recent patient-derived GB cells (PDC) cultured in defined media. These inducers and effectors of GB cell motility are involved in pro-motile autocrine signaling (TGFB1, SMAD3, THBS1 [29**–**31]), cell morphological rearrangements linking extracellular guidance signals with cytoskeleton remodeling (ACTN4 [32**–**34], PTK2 [35, 36], PXN [36**–**38], TLN1 [39], VCL [19]) and remodeling of the matri-cellular environment (TNC [35], SPARCL1 [40]). Expression of each signature gene is most often positively correlated with expression of the other signature genes across all cells (Fig. 1b, Table S1).

This clustering analysis resulted in six cell clusters (Fig. 1c) with distinct combinations of expression of the signature genes (Fig. 1d). Each cluster was composed of cells coming from distinct tumors, as indicated by the low Normalized Mutual Information value (NMI = 0.11; Fig. 1e). This demonstrates that cell clustering is driven by functional state rather than tumor of origin as we previously described [41]. Of note, HCPC integrating UMAP components led to groups of cells with transcriptomic profiles more homogeneous than when using HCPC alone, as shown by increased Silhouette width (Fig. S3B). Using the geometric mean of the signature gene expression levels as a motility score, we retained the two cell clusters with the highest (M^HIGH^) and lowest (M^LOW^) motile potential, respectively (Fig. 1f). Differential expression analysis between M^HIGH^ and M^LOW^ cell groups (Mann–Whitney, BH-adjusted *p*-value < 0.01) highlighted the genes overexpressed in M^HIGH^ cells (Table S2). Gene ontology (GO) enrichment analyses provided a first support to the suitability of our strategy for identifying motile cells by highlighting an enrichment of motility-related terms among the genes most overexpressed in M^HIGH^ cells (Fig. 1g and Table S3).

We next repeated the same analytical strategy with the three other selected datasets (Figure S3C–E). Each of the analyses resulted in clusters composed of cells coming from different tumors (NMI = 0.058–0.15; Fig. S3C–E). Comparing the lists of genes overexpressed in M^HIGH^ cell group between the datasets showed the highest overlaps between lists from datasets obtained with the same sequencing technology (69.2% (4852/7010) and 86.0% (6627/7707) between datasets obtained with SMART-seq2 and 10X Genomics technologies, respectively, Fig. S3F). Comparing the four lists of overexpressed genes showed that 43 to 53% of the genes overexpressed in M^HIGH^ cells from one dataset were also overexpressed in the three other datasets (Fig. S3F and Table S2). Finally, GO enrichment analyses of the most overexpressed genes, again highlighted enrichment in motility-related terms in each dataset (Fig. S4 and Table S3). These results indicate that our analytical strategy captures cells with similar transcriptional profiles enriched in motility-related terms in all datasets studied.

To further probe the suitability of our analytical approach for identifying cells in a motile state, we selected three gene modules previously found to be associated with GB cell motility (Table S2), and determined their enrichment in M^HIGH^ cells. Most motile cells, including GB cells, have been shown to undergo transcriptional reprogramming akin to the so-called epithelial-mesenchymal transformation (EMT) process. We therefore used an EMT gene module of 14 genes associated with mesenchymal transformation of neural cells and GB cell motility [19, 42]. We also sought for enrichments in two other gene modules. One consists of 36 genes with highly correlated expression in the outer radial glia (oRG)-like malignant cell population with increased invasive behavior in GB [10]. The other consists of 32 genes involved in the regulation of GB cell motility and driven by TEAD transcription factors [43]. Each of these gene modules was enriched in M^HIGH^ cells in all analyzed datasets (Fig. 1h). We also observed a positive linear relationship between the motility score and the EMT, oRG, and TEAD scores (Figs. 1i and S5). As expected from these correlations, the EMT, oRG, and TEAD scores were significantly higher in M^HIGH^ cells compared to M^LOW^ cells (Figs. 1j and S5).

Altogether, these results support the relevance of the analytical strategy implemented for capturing cells with high motile potential from distinct datasets.

### Metabolic reprogramming of cells with high motile potential

**Fig. 2:**
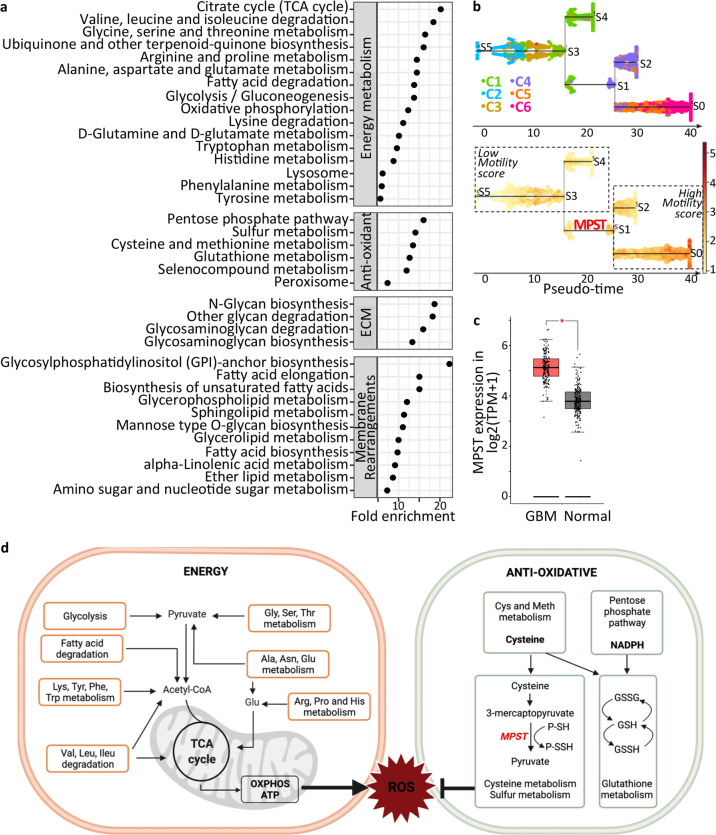
Metabolic reprogramming of cells with high motile potential. **a** KEGG pathway analysis of metabolism genes overexpressed in M^HIGH^ cells versus M^LOW^ cells highlights enrichment in pathways involved in energy production, oxidative stress response, extracellular matrix (ECM) modeling, and membrane composition rearrangements. BH-adjusted *p*-value < 0.05. N-S dataset analysis. **b** Inferred trajectory of GB cells from low to high motility. Cells colored by motility clusters (top panel) and motility score (bottom panel). MPST marks the path crossroad between low and high motile potential. N-S dataset analysis. **c** MPST overexpression in GB tissues (*n* = 163) versus normal brain tissues (*n* = 207). TCGA RNA-seq dataset, GEPIA2 website. One-way ANOVA test, *BH-adjusted *p*-value < 0.05. **d** Schematic representation of the metabolic pathways overrepresented in M^HIGH^ cells compared to M^LOW^ cells (created with BioRender.com). See also Tables S4 and S5.

We went on to identify metabolic pathways deregulated in GB cells with high motile potential. The genes coding for enzymatic components of the metabolic pathways were identified among the genes overexpressed in M^HIGH^ cells in each dataset using the 2019 KEGG list of 1586 metabolic elements [44] (Table S4). KEGG pathway enrichment analyses highlighted metabolic pathways related to ECM modeling (e.g. glycosaminoglycan synthesis/degradation) and membrane composition rearrangements (e.g. unsaturated fatty acids (FA), FA elongation, GPI-anchor biosynthesis; Fig. 2a and Table S4). In addition to these metabolic pathways expected to be active in motile cells, we also observed enrichment in pathways involved in energy production (Fig. 2a and Table S4). Overexpression of genes involved in glycolysis, amino acid catabolism, FA degradation, protein recycling (lysosome), TCA cycle, oxidative phosphorylation, suggests that M^HIGH^ cells have higher energetic needs than M^LOW^ cells. Enrichment in oxidative phosphorylation, a term gathering elements of the electron transport chain (ETC), associated with enrichment in TCA cycle components, suggests in addition a higher mitochondrial load in M^HIGH^ cells. Finally, we observed enrichment in pathways involved in anti-oxidative processes, such as the pentose phosphate pathway, and the cysteine, sulfur, and glutathione metabolisms (Fig. 2a and Table S4). Enrichments in pathways or organelles involved in reactive oxygen species (ROS) scavenging and/or production of anti-oxidants (porphyrin and selenocompound metabolisms, peroxisome), as well as enrichment in purine/pyrimidine metabolism required for DNA/RNA repair, are also compatible with enhanced responses to oxidative stress. Overall, the results of this analysis point to an enhanced mobilization of anti-oxidative pathways counteracting the deleterious actions of ROS derived from the over-mobilization of energy production pathways.

Trajectory modeling was used next to pinpoint metabolic components potentially crucial for acquiring a motile cell state. Trajectory reconstruction based on expression of the motility signature genes was achieved with the STREAM python package using the N-S dataset which has the highest sequencing depth. The inferred trajectory corresponded to a branched path, highlighting an intermediary branch (S3-S1 branch) linking cells with low motile potential to cells with high motile potential (Fig. 2b). Among the metabolic enzymes overexpressed in S3-S1 branch compared to each of the other branches, characterizing therefore the switch between low and high motile states, two were overexpressed in M^HIGH^ cells of all datasets considered, *NIT2* and *MPST* (Table S5). Their participation in the control of GB cell properties is unknown. We selected MPST for further studies owing to its potential anti-oxidative activity. MPST converts 3-mercaptopyruvate, derived from cysteine transamination, to pyruvate while transferring a sulfur to a thiophilic acceptor, thereby forming a persulfide [45]. It is thus endowed with potential anti-oxidant activities through protein persulfidation (P-SSH, also known as S-sulfhydration), a post-translational modification of reactive cysteine residues preventing their irreversible hyperoxidation [45, 46]. Interestingly, we observed MPST overexpression in GB tissues compared to normal brain tissues (Fig. 2c). In addition, we identified the mitochondria-located GOT2, an aminotransferase ensuring the formation of the MPST substrate 3-mercaptopyruvate, among the genes overexpressed in GB cells with high motile potential (Table S2).

Altogether, our computational analyses predict that enhanced oxidative stress coupled with MPST mobilization is instrumental in GB cell motility (Fig. 2d).

### Enhanced ROS production and mitochondrial mass characterize motile cells

We next verified experimentally whether enhanced oxidative stress and MPST mobilization were indeed functionally involved in GB cell motility. We first determined whether ROS affect GB cell motility, using GB patient-derived cells (PDC) or -derived tissue organoids (GBO) cultured in serum-free media. PDC relative migratory and invasive properties, determined using spheroid-on-Matrigel migration assays, invasion-across-collagen assays and Matrigel-coated transwell assays, showed 5706**-PDC to be the most motile, followed by R633- and P3-PDC (Fig. S6A–C).

**Fig. 3:**
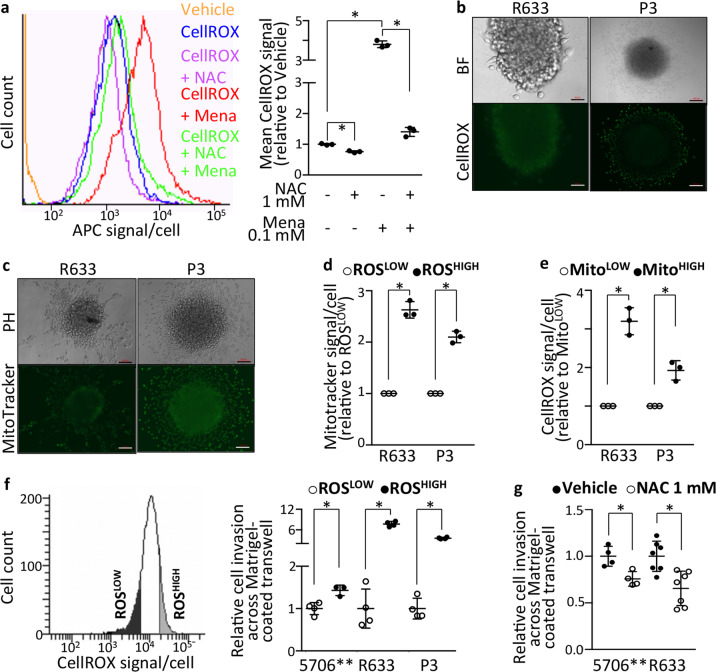
Enhanced ROS production and mitochondrial mass characterize motile cells. **a** Decreased CellROX Deep Red fluorescent signal in PDC treated with the anti-oxidant NAC (1 mM, 1 h) and increased signal in PDC treated with the ROS generator menadione (Mena, 0.1 mM, 30 min). 5706**-PDC. Mean ± SD, *n* = 3 independent biological samples, *p < 0.05, unpaired *t*-test with Welch’s correction. **b** Higher ROS production in cells migrating out of spheroids. Brightfield (top panel) and CellROX Green signal (488 nm, bottom panel) imaging. Scale bars = 50 µm (R633) and 200 µm (P3). **c** Higher mitochondrial mass detected with MitoTracker Green reagent in cells migrating out of spheroids. R633- and P3-PDC. Phase contrast (top panel) and MitoTracker signal (488 nm, bottom panel) imaging. Scale bars = 200 µm. **d**, **e** Higher mitochondrial mass in cells with high ROS production (**d**), and higher ROS production in cells with high mitochondrial mass (**e**). R633- and P3-PDC. FACS analysis. Mean ± SD, n = 3 independent biological samples, *p < 0.05, unpaired *t*-test with Welch’s correction. **f** Higher invasive properties of cells with high ROS production. 5706**, R633 and P3 PDC. Left panel: example of FACS-sorting of PDC into ROS^LOW^ and ROS^HIGH^ fractions. Right panel: cell invasion across Matrigel-coated transwells, mean ± SD, *n* = 3–4 independent biological samples, *p < 0.05, unpaired *t*-test with Welch’s correction. **g** Decreasing ROS levels in GB-PDC using 1 mM NAC decreases cell invasion. 5706** and R633 PDC. Cell invasion across Matrigel-coated transwells, mean ± SD, *n* = 4–7 independent biological samples, *p < 0.05, unpaired *t*-test with Welch’s correction. See also Fig. S6A–C.

Intracellular ROS levels were evaluated with CellROX^®^ Green or Deep red fluorogenic probes. Their suitability for detecting ROS in GB PDC was verified by FACS following PDC treatment with the anti-oxidant N-Acetyl Cysteine (NAC) or the ROS generator menadione (Fig. 3a). Microscopic observation of PDC-spheroids first seeded on Matrigel and then labeled with CellROX^®^ probe showed a higher ROS signal in GB cells migrating out of the spheroids compared to their static counterparts (Fig. 3b).

Considering this result with our observation of enriched expression in genes involved in the TCA cycle and the ETC in M^HIGH^ cells, we measured mitochondrial mass using the MitoTracker probe. An increased mitochondrial load was detected in cells migrating out from PDC-spheroids (Fig. 3c). FACS was then used to isolate PDC according to their CellROX or Mitrotracker signals. Cells with high ROS production exhibited a 2.1–2.6 times higher mitochondrial mass than cells with low ROS production (Fig. 3d). Conversely, cells with high mitochondrial mass exhibited a higher ROS production than cells with low mitochondrial mass (1.9–3.2 times more) (Fig. 3e). We next assessed the invasive potential of GB PDC with high ROS production. To do so, we FACS-sorted GB PDC according to their ROS production (Fig. 3f). We found a 1.4–7.7-fold increase in invasiveness of GB cells with high ROS levels compared to cells with low ROS levels, as determined with Matrigel-coated transwell invasion assays (Fig. 3f). In a coherent manner, decreasing ROS levels with NAC decreased GB cells’ invasiveness (Fig. 3g). These results indicate that motile GB cells exhibit enhanced ROS production, as predicted by the results of the computational analyses, and that ROS participate to GB cell motility.

### MPST enzymatic activity is required for GB cell motility

**Fig. 4:**
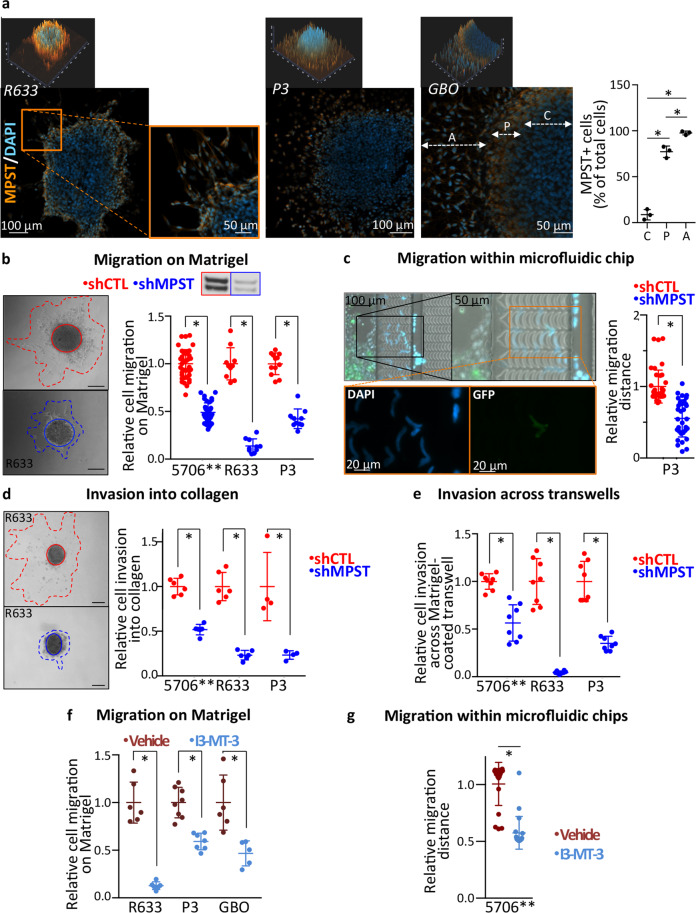
MPST enzymatic activity is required for GB cell motility. **a** MPST immunolabeling (orange) of GB cells migrating out of PDC spheroids or GBOs. Nuclei DAPI staining in blue. Upper images illustrate the 2.5D intensity plot of DAPI (blue) and MPST (orange) signals across and around spheroids during migration on Matrigel (ZEN software, Zeiss). Individual peaks represent absolute signal intensities of each pixel. Graph: quantification of the percentage of MPST-immunoreactive cells in PDC-spheroids and GBO within the spheroid core (C), at the spheroid periphery (P), and away from the spheroids (A), as indicated with the dotted double arrows in the adjacent microphotograph. Mean ±SD, n = 3, *p < 0.05, Newman-Keul multiple comparisons test. **b** *MPST* knockdown decreases cell migration on Matrigel. Microphotographs illustrate cell migration assays, scale bars = 200 µm, solid line = spheroid core, dotted line = migration area. Graph: quantification at 7 h (5706**) or 24 h (R633 and P3) post-seeding, mean ± SD, *n* = 10-41 independent biological samples, Mann–Whitney test, *p < 0.05. Inset above graph illustrates decreased MPST protein levels in sh*MPST*-PDC (western blot analysis, MW: 33/35 kDa). **c** *MPST* knockdown impairs cell migration through microfluidics chip tortuous channels. P3-PDC co-expressing sh*MPST* and GFP were loaded together with equal numbers of shControl-PDC. Only rare sh*MPST*-PDC (green cells) cross the microchip channels, contrary to shControl-PDC (DAPI^+^/GFP^-^). Scale bar = 200 µm. Graph: distances traveled by the cells over 24 h, mean ± SD, *n* = 32–40 from 4 independent biological samples, *p < 0.05, Mann–Whitney test. **d** *MPST* knockdown decreases cell invasion into collagen. Microphotographs illustrate invasion assays, scale bars = 200 µm. Graph: quantification of the invasion after 24 h (R633- and P3-PDC) or 40 h (5706**-PDC), mean ± SD, *n* = 4–8 independent biological samples, *p < 0.05, Mann–Whitney test. **e** *MPST* knockdown decreases cell invasion across Matrigel-coated transwells. 5706**-, R633- and P3-PDC invasion assessed 24 h post-seeding, mean ± SD, *n* = 4–8 independent biological samples, *p < 0.05, Mann–Whitney test. **f** Inhibiting MPST enzymatic activity using the pharmacological inhibitor I3-MT-3 decreases cell migration on Matrigel. Cells treated with 200 µM I3-MT-3 or vehicle for 72 h (R633, P3) or 45 h (GBO). Migration assessed 48 h (R633, P3) and 45 h (GBO) post-seeding. Mean ± SD, *n* = 5–8 independent biological samples, *p < 0.05, Mann–Whitney test. **g** Inhibiting MPST enzymatic activity with I3-MT-3 decreases cell migration into microfluidic chips. Graph: greatest distances traveled by the cells 24 hr post-seeding. 5706**-PDC, mean ± SD, *n* = 20 from two independent biological samples, *p < 0.05, Mann–Whitney test. See also Figs. S6D-I and S7.

To determine if MPST is necessary for GB cell motility, we first assessed MPST protein levels in motile GB cells by immunocytochemistry. As expected, the profile of the MPST-immunoreactive signal overlapped with those of the CellROS and MitoTracker signals (Fig. S6D). The highest MPST-immunoreactive signal was observed in cells located at the periphery of PDC-spheroids and of tissue organoids (GBO), and in cells that migrated out of the spheroids or of GBO (Fig. 4a). In coherence with the results of the bioinformatics analyses predicting enhanced MPST expression in migrating cells, quantification of MPST-immunoreactive cells confirmed that the proportion of cells expressing MPST was far greater in cells located away from the spheroids (97.6 ± 2.2%) and at its periphery (77.2 ± 6.2%) than within the spheroid cores (8.7 ± 5.7%) (Fig. 4a, graph). We next knocked down *MPST* expression using lentiviral transduction of a small hairpin (sh) RNA, which led to decreased MPST mRNA and protein levels with no major cytotoxic effect (Figs. S6E–G and S7). Similar results were obtained with another sh*MPST* construct (Fig. S6I, J). Evaluation of the overall energetic metabolism using the Seahorse XFe24 Analyzer showed no major change in mitochondrial respiration upon *MPST* knockdown (Fig. S6H). Knocking down *MPST* expression resulted in a sharp decrease in cell migration (51-86%) as determined with spheroid-on-Matrigel assays (Figs. 4b and S6K). Effect of MPST knockdown on cell migration was also probed using microfluidic chips, which allow mimicking the constrained space through which the cells move within tissues (further described in supplemental methods). Monitoring cells’ ability to move across 4.5-µm-height curved micro-channels with periodic 3 µm mechanical constraints in a microfluidic chip further showed that *MPST* knockdown also impaired GB cells’ ability to change their shape so as to migrate through narrow and tortuous spaces (Fig. 4c). Cell invasion was also decreased by 44–95% upon *MPST* knockdown as observed using spheroid-in-collagen (Fig. 4d) and Matrigel-coated transwell invasion assays (Fig. 4e).

**Fig. 5:**
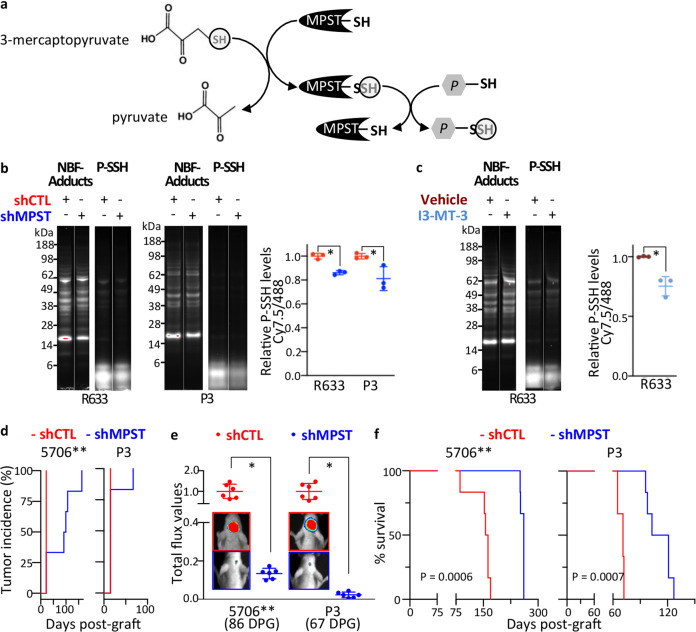
*MPST* knockdown decreases protein persulfidation, decreases tumor burden, and increases mice survival expectancy. **a** Schematic representation of MPST catalytic activity, with the successive transfer of the SH group from its substrate 3-mercaptopyruvate, first to the enzyme itself, and then to protein (P) cysteine residues. **b** Decreased protein persulfidation (P-SSH) levels in sh*MPST*-PDC vs shControl-PDC. R633 and P3 PDC. In-gel detection of persulfidation levels. Dimedone switch method with Cy7.5 as a P-SSH reporting molecule. P-SSH levels calculated as a ratio of Cy7.5/NBF-protein adducts signal (488 nm), mean ± SD, *n* = 3 independent biological samples, *p < 0.05, unpaired *t*-test. **c** Decreased protein persulfidation levels upon cell treatment with the MPST inhibitor I3-MT-3. R633 PDC. In-gel detection and calculation of P-SSH levels as in **b**. Mean ± SD, *n* = 3 independent biological samples, *p < 0.05, unpaired *t*-test. **d** *MPST* knockdown does not prevent tumor initiation. Bioluminescent analyses. 5706** and P3 PDC. n = 6 mice per group. DPG: days post-graft. **e** *MPST* knockdown decreases tumor burden as shown by quantification of the tumor bioluminescent signals. Mean ± SD, *n* = 6 mice per group, *p < 0.01, Mann–Whitney test. See also Fig. S6L. **f**. *MPST* knockdown results in a significant survival benefit. Kaplan–Meier survival curves, *n* = 6 mice per group, Log-rank (Mantel-Cox) test.

The observed decrease in cell motility can be due to an inhibition of MPST catalytic activity or to defects in other MPST functions. To distinguish between these two possibilities, we treated cells or GBOs with 200 µM I3-MT-3, a selective pharmacological inhibitor of MPST enzymatic activity [47]. I3-MT-3 decreased PDC migration out from PDC-spheroids and GBO seeded on Matrigel (Fig. 4f). I3-MT-3 inhibited also PDC migration through constraining microfluidic chip channels (Fig. 4g). These results indicated that MPST catalytic activity was required for GB cell motility. Mechanistically, MPST catalyzes a persulfidation process through a two-step transfer of the SH group from its substrate 3-mercaptopyruvate, first to the enzyme itself, and then to protein cysteine residues and other molecules resulting in their persulfidation [45] (Fig. 5a). We therefore determined protein persulfidation levels in PDC with decreased MPST expression or activity using the recently published Dimedone-Switch method [48]. NBF-Cl protein labeling showed similar protein patterns in control PDC and in PDC expressing sh*MPST* or treated with I3-MT-3 (Figs. 5b, c and S8). In control PDC, persulfidated proteins were detected within both high and low molecular weight ranges, albeit at higher levels at low molecular weights. *MPST* knockdown, as well as MPST enzymatic inhibition, resulted in an overall decrease in protein persulfidation levels across all molecular weight ranges (Fig. 5b, c and S8). These results show that in GB cells, MPST mechanism of action implicates protein persulfidation.

Finally, we evaluated the consequences of *MPST* knockdown on overall tumor burden using brain xenografts of PDC stably expressing luciferase and either shControl or shMPST. Bioluminescence imaging showed tumor initiation from shControl and shMPST-expressing cells (Fig. 5d). We observed a reduced tumor burden in mice grafted with sh*MPST*-PDC, compared to mice grafted with shControl-PDC up to the experiment end-points (Figs. 5e and S6L), and a 1.6-fold increase in the median survival expectancy of mice xenografted with shMPST-PDC (Fig. 5f).

## Discussion

The diversity of cancer cells populating GB is a major challenge in treating these brain cancers. Understanding the molecular basis of the varying functional cell states co-existing within GB at the time of patients’ diagnosis and commonly shared across tumors, opens a path to overcome this challenge. A way to access such information is coupling experimental validations with computational modeling of transcriptomes of single cells sorted from patient GB, which offer one of the closest possible maps of the patients’ tumors. Following this blueprint led to circumscribing the overall metabolic characteristics of motile cells across thirty patients’ GB, and to identifying MPST as a metabolic enzyme crucial for GB cell motility and tumor development.

GB cells with high motile potential were characterized by higher energetic production than cells with low motile potential, as shown by the enrichment in multiple energetic pathways. This is in agreement with motile cell needing to mobilize different energetic pathways to adapt to new microenvironments with unique nutrient and oxygen availability [49]. The predicted convergence of this overall energetic mobilization towards the TCA and the ETC was verified by the experimental demonstration of enhanced ROS production and higher mitochondrial mass load in motile GB cells. These results are coherent with the reported stimulation of direct redox modification of protein components of pro-migratory signaling pathways [50, 51], and with increased mitochondrial superoxide production during epithelial cancer cell metastasis [52].

Intracellular ROS levels must however be tightly controlled since ROS overload can have severe deleterious consequences on cell properties and viability. Our results indicate that GB cells with high motile potential are enriched in anti-oxidant pathways capable of counteracting oxidative stress including the pentose phosphate pathway, which implicates enhanced NADPH production necessary for the function of several antioxidant proteins, and the glycine, glutamate and cysteine metabolisms together with glutathione metabolism that implicates enhanced availability of the major anti-oxidant compound GSH. Cysteine metabolism involvement in the anti-oxidative process extends beyond supporting GSH synthesis. The enzymes of the cysteine metabolism, Cystathionine Gamma-Lyase (CGL or CSE, encoded by *CTH*), Cystathionine Beta-Synthase (CBS) and MPST (also known as MST), participate to protein protection from detrimental hyperoxidation of their reactive cysteine residues [48].

MPST was the only metabolic enzyme participating in protection from oxidative stress predicted by trajectory modeling to be at the crossroad of the path leading GB cells from low to high motility. Our results demonstrate the biological validity of this prediction. MPST knockdown resulted in decreased ability of the cells to move around in a freely available space, as well as to move into their surroundings by remodeling the ECM. Interestingly, MPST has been linked to the migration of murine colon cancer cell line CT26 [53], raising the possibility of its involvement in the motility of cancer cells other than GB cells. Alteration of GB cell properties by MPST knockdown translates in vivo into reduced tumor burden, and a robust increase in mice survival, albeit the cells retain their tumor-initiating properties. MPST enzymatic activity is required for its pro-motile effects, the MPST pharmacological inhibitor I3-MT-3 [47] having the same consequences as MPST knockdown on GB cell migration. Protein persulfidation is part of the mechanisms by which MPST affects GB cell motility. Reduced MPST expression or activity was accompanied with decreased persulfidated protein levels in GB cells.

A few surveys of persulfidated proteins have been performed in control conditions, without dihydrogene sulfide (H_2_S) supplementation [48, 54–57]. Interestingly, proteins pertaining to the migration machinery and associated signaling pathways were found to undergo persulfidation, such as the focal protein kinase encoded by *PTKN2* and EGFR in EGF-treated HeLa cells [48], or actin in the mouse liver [56]. The overall protection of proteins from hyperoxidative damage conferred by this post-translational modification can also be accompanied with more specific consequences. Persulfidation was reported to enhance actin polymerization and alter actin-dependent cytoskeletal rearrangements in HEK293 cells [56], and the persulfidated form of glycolytic enzymes such as GAPDH or LDHA were found to exhibit enhanced catalytic activity [55, 56, 58]. The protein persulfidations reported in these studies have been associated with CBS and/or CSE activities, as supported by studies implementing inactivation of these enzymes and/or by their conspicuous expression in the studied biological systems [48, 55–59]. In addition to producing low molecular weight persulfides [59**–**61], MPST has also been found to mediate protein persulfidation of the sulfurtransferase Mocs3 and of thioredoxin (Trx). Through Mocs3, MPST is involved in a cascade leading to protein urmylation and tRNA thiolation, and through Trx in H_2_S generation [45]. Our observation of an overall decrease in protein persulfidation levels upon MPST knockdown indicates that MPST can affect a wider range of proteins at least in GB cells. Identifying the specific targets of MPST in GB motile cells will require further studies combining selection of motile and static cells with immunoprecipitation methods coupled with proteomic profiling.

Whether our results pertain to a specific mode of cell motility and/or path of migration calls for further investigations. Enrichment of oRG-like gene modules in M^HIGH^ cells suggests that the motile cells we captured move in a solitary rather than in a collective fashion, like oRGs in the developing cortex. The observation that cells expressing neutral shRNA or shMPST maintain their respective motile or non-motile properties when mixed in equal numbers in microfluidic chip assays also supports a cell-autonomous action of MPST. The cells analyzed in our study most likely come from dense tumor cell regions since removal of the surrounding parenchyma is undertaken with great caution by neurosurgeons in order to spare at the best motor and cognitive functions. Our findings are therefore likely to relate to cells moving along the blood vessel network that characterize these highly angiogenic tumors, or across the tumor parenchyma enriched in components favoring cell displacement.

Discovery of the participation of MPST in the control of GB cell motility was made possible by considering metabolism in the context of its cell-by-cell variability in the human tumor. Altogether, our results highlight a new face of oxidative stress mechanism of action in promoting malignancy by unraveling the essential role of the MPST enzyme in cell motility, and thereby a metabolic dependency shared by tumors with variable genomic landscapes. They thus provide an important step forward in our understanding of the metabolic elements critical for cancer cell motility.

## Material and methods

Table S6 lists all resources, materials, bio-informatics tools, corresponding websites and references. Cell imaging was achieved with a fluorescent microscope Axioplan 2 (Zeiss).

### Computational analyses

Of the publicly-available single-cell transcriptomic datasets from GB tumors collected from adult patients, we downloaded the only two obtained with the SMART-seq2 technique [9, 27], and two of the ones obtained with the 10X Genomics technique [9, 28] (Table S6). The latter were selected over others because of the raw UMI counts availability. Malignant and normal cells were distinguished either according to the cell annotations provided for both SMART-seq2 datasets, or when absent as for 10X Genomics datasets, based on inference of copy-number variations (CNVs), a hallmark of malignant cells (Supplemental methods, Figs. S1 and 2). Data pre-processing is further detailed in Supplemental methods. As a result, we retained for further analyses 4916 malignant cells from twenty GB in the N-S dataset, 1033 from four GB in the D-S dataset, 5797 from six GB in the N-10X dataset and 8666 from six GB in the PA-10X dataset. Log_2_-transformed Counts Per Million (log_2_(CPM + 1)) were used for D-S and the 10X datasets, and log_2_-transformed Transcripts Per Million (log_2_(TPM + 1) for N-S dataset, unless otherwise specified (Supplemental methods). To avoid potential analytical bias due to scarcely detected genes, genes detected in less than 1% cells were filtered out.

Cell grouping analyses based on a molecular signature of ten elements were achieved using the HCPC approach (FactoMineR package [62]) modified to implement also UMAP components (Supplemental methods, Fig. S3A). The cell grouping was visualized using UMAP, chord plots or heatmaps with optimized color palettes (Table S6). NMI values were calculated to determine the contribution of cells issued from distinct tumors to each cluster (Supplemental methods). Motility score was obtained by computing the geometric mean of the expression values per cell of each element of the molecular signature. When null, expression values were imputed with a value of 1.

Genes differentially expressed between cell groups with differing scores were identified using Mann–Whitney test (Benjamini–Hochberg (BH)-adjusted *p*-value < 0.01). Genes coding for metabolism enzymes were identified using the list from KEGG. Comparing lists of differentially expressed genes between distinct datasets was performed on genes detected in all datasets (eulerr package). Gene ontology analyses were carried out on the enrichR website. Significance level was set at BH-adjusted *p*-value < 0.05 (Fisher’s Exact test). Redundant terms were excluded. Significance level was set at BH-adjusted *p*-value < 0.05 (Fisher’s Exact test). Graphs were generated using ggplot2 R package.

The path taken by cells with low motile potential to reach a high motile potential was modeled using trajectory inference analysis (STREAM python package [63]). Briefly, the expression values of the ten elements of the motility signature were extracted, and dimensions reduced to four components using the spectral embedding algorithm. The components were then used for simultaneous tree structure learning and fitting using ElPiGraph. The resulting trajectory structure was represented in a 2D subway map plot, where straight lines represent branches and each dot represents a single cell. Genes differentially expressed between cell populations from two adjacent branches were identfied (Mann–Whitney *U* test, *q*-value < 0.05).

Supplemental methods describe further methodological details and datafiles S1 and S2 the R and Python scripts used.

### Biological material, lentiviral transduction and pharmacological treatment

PDC-R633, -5706** and -P3 obtained in our and other laboratories from neurosurgical biopsy samples of distinct GB were cultured in defined medium containing bFGF and EGF or bFGF only (P3), as described [64**–**66]. They stably express luciferase, and 5706**-PDC express GFP as well. Lentiviral vectors were used to transduce cells with a shRNA either neutral (shControl) or targeting *MPST* transcripts (sh*MPST*; Table S6). The shMPST construct encoding GFP in addition to the shRNA allowed distinction between shControl and shMPST cells. Non-transduced cells were eliminated with puromycin (1–2 µg/mL). GBO were generated from a GB patient tumor (Table S6) as described [67]. In relevant experiments, cells were treated with 200 µM I3-MT-3 (MedChemExpress) or 1 mM NAC (Sigma) or their vehicles (DMSO and culture media respectively).

### Cell motility assays

Cell migration was assessed using spheroid-on-Matrigel assay, as described [68], and microfluidic chips. The chips were designed and fabricated, as described [69], and included successive mechanical constraints for assessing cells’ ability to change shape so as to move through narrow and tortuous spaces. Cell invasion was assessed using spheroid-in-collagen assay and Matrigel-coated transwells as described [68, 70]. Further details and quantification methods are given in Supplemental methods.

### Detection of intracellular ROS levels

Cells or spheroids were incubated at 37 °C for 30 min with CellROX Green (5 µM/R633, 10 µM/P3) or Deep Red reagent (1.25 µM/P3, 2.5 µM/R633 and 5706**) (ThermoFisher). Fluorescence signal was then assessed by microscopy (CellROX Green, Zeiss microscope) or FACS (CellROX Deep Red, ARIA II, BD Biosciences). When indicated, cells were treated with NAC (1 h, 37 °C) and/or Menadione (30 min, 37 °C), prior FACS analysis of ROS levels as detailed in Supplemental methods.

### Measure of mitochondrial mass

Cells, spheroids, or GBO were incubated with 1 µM MitoTracker Green reagent (Invitrogen) at 37 °C for 30 min and the fluorescence signal was assessed by microscopy or FACS (Supplemental methods).

### Gene and protein expression analyses

Gene expression knockdown in response to shRNA expression was verified by RT-QPCR as previously described [41] using the LightCycler480 (Roche, France) and the SYBR Green PCR Core Reagents kit (Bimake.com; primer sequences in Table S6).

MPST protein levels were determined using immunobloting assays with anti-MPST (Sigma, 1:4000), and ImageJ software for densitometric analysis. MPST signals were normalized by the amount of protein load as assessed with Red Ponceau. For immunocytochemistry, harvested **c**ells were PBS washed, smeared on SuperFrost slides (ThermoScientific, France), fixed with 4% paraformaldehyde, PBS washed and incubated in Antibody blocker/diluent (Enzo, 1 hr, room temperature). MPST antibody (Sigma, 1:50, overnight, 4 °C) and next anti-rabbit A555 (Life Technologies, 1:500, 1 h, room temperature) were applied. Fluorescent signals were analyzed after nuclear DAPI staining (Sigma) with Zen software (Zeiss) using 2.5 µm optical sections.

Persulfidated protein levels were assessed following the Dimedone-Switch protocol, as described by Zivanovic and colleagues [48] (Supplemental methods). Briefly, this method relies on the indiscriminate labeling of persulfides, thiols, sulfenic acids, and amino groups with the fluorogenic 4-chloro-7-nitrobenzofurazan (NBF-Cl, Em = 488 nm). Subsequent incubation with a Cy7.5-tagged dimedone derivative that reacts selectively with the persulfides, enables persulfide identification by Cy7.5-labeling. The NBF-Cl (Em = 488 nm) and Cy7.5 (Em = 800 nm) signals are recorded in gel after protein separation (Chemidoc Imager, BioRad, France). The Cy7.5 persulfidation signals were normalized by the A488 (NBF-adducts) signals.

### Animals

Animal experimentation was approved by Comité d’éthique en expérimentation animale Charles Darwin No. 5 (Protocol #5379). In all, 1.5 × 10^5^ cells were injected stereotaxically into the striatum of anesthetized 8-10 weeks-old Nude female mice (Janvier Laboratories, France), and tumor formation and development monitored by bioluminescence imaging (Biospace Lab, M3 Vision software, France) as described [41].

### Statistical analyses

R or Prism 7.0 (GraphPad) softwares were used to generate plots and for statistical analyses. Significance level was set at *p* < 0.05, unless otherwise indicated. Each statistical test used is provided in the figure legends. All experiments were performed using independent biological samples. Mean ± SD are shown.

## Supplementary information

Supplemental methods and legends

Supplemental Figures

Supplemental Table S1

Supplemental Table S2

Supplemental Table S3

Supplemental Table S4

Supplemental Table S5

Supplemental Table S6

Datafile S1

Datafile S2

Reproducibility Checklist

## Data Availability

All data generated during this study has been included in this manuscript and its supplemental files.
