# Supplementary material for: Glioblastoma cell motility depends on enhanced oxidative stress coupled with mobilization of a sulfurtransferase
Source: Cell Death Dis. 2022 Oct 30;13(10):913. doi: 10.1038/s41419-022-05358-8 (PMC9618559; doi:10.1038/s41419-022-05358-8)
Supplement: Supplementary file 1 — Supplemental methods and legends [file 41419_2022_5358_MOESM1_ESM.docx]

**SUPPLEMENTAL METHODS AND LEGENDS**

**Supplemental methods, related to methods**

**scRNA-seq data pre-processing**

Outlier cells with low-complexity transcriptomes, revealed by plotting the graphical distribution of the number of detected transcripts and genes per cell, were excluded from the analyses as described (1). For 10X datasets, detection of over 20% mitochondrial genes per cell was considered as indicative of dying cells leading to their exclusion from the analyses. Therefore, we retained for further analyses 4916 malignant cells from twenty patients in the N-S dataset, 1033 malignant cells from four patients in the D-S dataset, 5797 malignant cells from six patients in the N-10X dataset and 8666 malignant cells from six patients in the PA-10X dataset. They are designated as N-S, D-S, N-10X and PA-10X, according to the initials of the first author of the paper reporting its first description and the technology used.

Log2-transformed Counts Per Million (log2(CPM+1)) were used for D-S and the 10X datasets, unless otherwise specified. CPM corresponds to the counts of gene-mapped reads normalized by the total number of mapped reads per cell divided by one million, thus allowing comparison of read abundance across libraries of different sizes. For the N-S dataset, gene expression data currently available are in log2((TPM/10) + 1). TPM (Transcripts Per Million) normalization takes into account an eventual biased estimation of long transcript numbers, by dividing the number of mapped reads by the transcript’s length. Since expression values in CPM could not be calculated from these data, log2((TPM/10) + 1) values were just transformed into log2(TPM + 1) to optimize the comparison between the different datasets. To avoid potential analytical bias due to scarcely detected genes, genes detected in less than 1% cells were filtered out.

**Identification of malignant and normal cells**

Malignant and normal cells were distinguished either according to the cell annotations provided for both SMART-seq2 datasets, or when absent as for 10X Genomics datasets, based on inference of copy-number variations (CNVs), a hallmark of malignant cells. Expression data in log2(CPM/100 + 1) values were used, which shrinks expression ranges, allowing to keep only the major gene expression variations, based on which malignant and normal cells can be distinguished. Data were processed using a three-step approach: CNV inference, marker gene expression and unsupervised cell clustering. CONICSmat R package used to infer CNVs has the advantage of not requiring an a priori reference cell dataset (2). The default filtering and normalization procedures were followed, as outlined in https://github.com/diazlab/CONICS/wiki/Tutorial---CONICSmat;-­‐Dataset:-SmartSeq2-scRNA-seq-of-Oligodendroglioma.

CONICSmat fits a two-component Gaussian Mixture Model (GMM) to the average gene expression across all cells within each chromosomal region. As a result, genes of a given region present a lower expression in cells with a deletion of this region than cells without the deletion. The opposite is observed for genes of amplified regions. The posterior probabilities of belonging to one of the two components of the model are then calculated for each cell. The copy number status across cells is predicted by the posterior probabilities for each cell belonging to the component with the higher mean. For each region, CONICSmat likelihood ratio test adjusted p-value <0.001 and a difference in Bayesian Criterion >300 were retained as CNVs. The GMM-based CNV predictions were then used to group cells into potential malignant and non-malignant groups and were visualized using heatmaps (ComplexHeatmap R package (3)) and UMAP (umap R package) plots based on 500 most variable genes (Supplemental figures 1A-B and 2A-B). Second, expression of marker genes for pan-immune cells (PTPRC/CD45), macrophages (ITGAM, FCGR3A/CD16A, CD14), microglia (CSF1R, TMEM119), T-cells (CD2, CD3D) and oligodendrocytes (MOG, MAG) was highlighted on UMAP plots (Supplemental figures 1C and 2C). Finally, a hierarchical clustering followed by a K-means clustering on the UMAP components (FactoMineR R package (4)) was applied to identify cell groups that were most similar or different to one another (Supplemental figures 1D and 2D). Using CNV status predictions, marker gene expressions and the clustering result, the cells were marked as malignant when they harbored CNVs, clustered together on UMAP and were devoid of normal cell markers (Supplemental figures 1E and 2E).

**Cell grouping analyses**

Clustering analysis was based on a molecular signature of ten elements (see results) and achieved using the Hierarchical Clustering on Principal Components (HCPC) approach (FactoMineR package (4) modified to implement, in a stepwise manner, Principal Component Analysis (PCA), Uniform Manifold Approximation and Projection (UMAP), hierarchical clustering, and partitioning clustering by the k-means (Supplemental Figure 3A). The ten principal components identified by PCA were reduced non-linearly to two dimensions using UMAP. Euclidean distance was used to construct a cell-to-cell distance matrix based on the two UMAP components. Hierarchical clustering was then performed on this distance matrix using the Ward’s criterion (ward.D2 algorithm) in order to determine the number of clusters. The resulting partitioning of the cells was improved by a K-means clustering with ten iterations. The cell grouping was visualized using UMAP (umap package) or chord plots (circlize package (5)) or heatmaps (ComplexHeatmap package (3)).

**Normalized Mutual Information (NMI) score calculation**

A Normalized Mutual Information (NMI) score (ClusterR package (6)) was calculated to determine the contribution of cells issued from distinct tumors to each cluster, as described (1). A NMI value of 1 implies that clusters gather cells corresponding to a single tumor, whereas a value of 0 denotes that each tumor contributes to each cluster.

**Differential expression analysis**

Genes differentially expressed between cell groups with differing scores were identified using a Mann-Whitney test with p-values adjusted for multiple testing (Benjamini-Hochberg (BH) adjusted p-value < 0.01) (7). Fold change (FC) was calculated as follows: FCi=xi−yi, where xi and yi are the log2 expression levels of gene i in conditions x and y, respectively. Only genes detected in ≥ 3% of GB cells were considered for this analysis. Gene ontology analyses were carried out on the enrichR website (8–10).

**Comparing lists of differentially expressed genes**

Comparing lists of differentially expressed genes between distinct datasets was performed on genes detected in ≥3% malignant cells in all datasets (eulerr package), and after updating gene symbols based on gene metadata files downloaded from HGNC and NCBI websites (Supplemental Table 6). Gene metadata file was downloaded from NCBI website using mimma R package (11). Genes absent from these metadata files, or with ambiguous symbols (e.g. genes whose current approved symbol is the previous symbol of another gene), or whose symbol was previously associated with more than one gene were excluded from the lists.

**Trajectory inference analysis**

To model the path taken by cells with low motile potential to reach a high motile potential, STREAM python package was used (12). Briefly, the expression values of the ten elements of the motility signature were first extracted. Next, dimensions were reduced to four components using the spectral embedding algorithm (dimension_reduction STREAM function, default parameters). The components were then used for simultaneous tree structure learning and fitting using ElPiGraph (seed_elastic_principal_graph STREAM function, default parameters). The resulting trajectory structure was represented in a 2D subway map plot, where straight lines represent branches and each dot represents a single cell (plot_stream_sc STREAM function, dist_scale = 0.5 and root = ‘S5’, other parameters set to default). To identify genes differentially expressed between cell populations from two adjacent branches (diverging genes), the detect_de_markers STREAM function was used. The mean of scaled gene expressions in each branch was calculated. Then, mean expression FC between pairs of branches was computed, and Mann-Whitney U test performed. The U statistic was then standardized to Z- score to assess in which branch the gene is overexpressed. Genes with Z-score greater than 1 and log2FC greater than 0.15 are considered as overexpressed between two adjacent branches. Multiple testing correction was performed using the Benjamini-Hochberg method. Significance level was set at q-value <0.05.

**Microfluidic chip fabrication**

Microfluidic chips were designed and fabricated, as described (13). Briefly, PDMS (Polydimethylsiloxane) blocks were modeled from a SU8 wafer encoding two 3mm-long 1mm wide and 50 µm-high cell culture chambers. The chambers are separated by 350µm-long, 4.5µm-high arrays of archlike microstructures encoding successive 3µm-width mechanical constraints that enforce cells to change their shape in order to move. Upon seeding in the cell culture chambers, the cells are pushed toward the microstructures, thanks to microfluidic pressure. The cells can then enter the microstructures that mimick the constrained space through which the cells move within tissues. The resulting SU8 wafers were used to cast polydimethylsiloxane (PDMS Sylgard, Ellsworth Adhesives) mixed at a 10:1 w:w ratio of base to curing agent. PDMS was cured 3 h minimum at 70 °C. The resulting chips were unmolded, and inlets were punched with a surgical biopsy punch (4 mm diameter) at both extremities of each compartment. The PDMS blocks were bonded to 130-160 µm thick glass coverslips (Fisher Scientific 11767065) after plasma surface treatment with an Atto plasma cleaner (Diener). Culture compartments were then immediately filled with deionized water. Devices were placed in individual Petri dishes for easier handling, and each of the resulting culture system was sterilized by exposure to a UV lamp for 30 min before use.

**Cell migration and invasion assays**

Cell migration was assessed using spheroid-on-Matrigel assay, as described (14). Briefly, cell spheroids were obtained by seeding 2500 cells/well into 100µL culture medium supplemented with 0.4% methylcellulose (Sigma) using 96- well U-bottom plates (Falcon). After a 24-48h culture, the cell spheroids were deposited on Matrigel (0.2mg/mL culture media, Corning). Cell migration was assessed 7h (5706**-PDC) or 24h (R633-, P3-PDC) later, unless specified otherwise. The migratory index was calculated as the ratio between the total area covered by GB cells migrating from a spheroid or GBO and the spheroid core or GBO area, as delineated and measured with FIJI software. Values were normalized to shControl or Vehicle. For microfluidic chip, 2*10^5^ PDC expressing shControl or sh*MPST* were seeded at a 1:1 ratio into the cell reservoirs. The sh*MPST* construct encoding GFP in addition to the shRNA allowed distinction between shControl and sh*MPST* cells. Seven days post-seeding, cells were fixed with 4% paraformaldehyde, and nuclei stained using DAPI (Sigma). The distance travelled by the cells that moved away from the channel entrance was quantified using FIJI software. To illustrate quantitatively the sharp difference between both cell populations, we measured all the rare shMPST-cells having moved away from the channel entrance, and selected equivalent numbers of shControl-cells among the ones having moved the furthest away. For I3-MT-3 tests, 2*10^5^ vehicle- or I3-MT-3-treated cells were seeded and cell migration quantified by measuring the distance traveled by the ten cells having migrated the furthest away from the channel entrance.

Cell invasion was assessed using spheroid-in-collagen assay (14) and Matrigel-coated transwells (15). Cell invasion was measured 24h after seeding spheroids generated as described above into collagen (1mg/mL culture media, Corning). The invasive index calculated corresponds to the ratio of the total area and the spheroid core area. Values were normalized to shControl or Vehicle. Transwells (8µm pores, Corning) were coated for 30min with Matrigel (0.2mg/mL) and placed in wells containing culture media with growth factors. 40,000 (R633-, P3-PDC) or 20,000 (5706**-PDC) cells in 100µL media without growth factors were seeded into the transwells. Cells having invaded the bottom part of the membrane were counted 24h later after paraformaldehyde fixation and DAPI-nuclei staining, as described (15).

**FACS analysis**

FACS analysis was performed on 10000-gated events using Vdye-labeled cells for detecting any non-viable cell (1µL/4-5*105 cells/mL, 30min, 4°C, eBiosciences). Violet laser (405nm) and Pacific Blue filter were used for Vdye detection, Blue laser (488nm) and FITC filter for CellROX Green or MitoTracker Green reagent detection, and Red laser (640nm) and APC filter for CellROX Deep Red reagent detection. Data analysis and figure generation were performed using the FACS Diva software (BD Biosciences).

**Seahorse bioenergetic analysis**

Energetic metabolism was analyzed on a Seahorse extracellular flux analyzer (XFe24) according to the manufacturer’s instructions (Agilent Technologies, France). PDC expressing either shCTL or sh*MPST* were seeded at a concentration of 1.2*10^5^ cells/well into Matrigel-coated Agilent Seahorse XFe24 (102340) microplates one hour prior analysis. The cells were then incubated in XF DMEM assay medium (Agilent Technologies, 103680-100) supplemented with 0.5mM sodium pyruvate (Gibco, 11360-070), 2mM glutamine (Life Technology, 25030024), and 18mM glucose (Gibco, A24940-01) for 1hr incubation in a CO_2_-free incubator at 37°C to allow temperature and pH equilibration. Oxygen Consumption Rate (OCR) was measured in basal condition (3 measurement cycles) and after sequential injections of oligomycin A (1 μM, 1 μM, SIGMA, 75351), FCCP (1 μM, SIGMA, SML2959-1ML) and a mix of rotenone/antimycin A (0.5 μM each, SIGMA, R8875 and A8674 respectively) with 6 measurement cycles after each injection and 3 final measurement cycles. Analyses were conducted using Seahorse Wave Controller Software v2.6 and XF Report Generators (Agilent Technologies). After Seahorse analysis, DNA content was measured using DAPI staining for normalization.

**RT-QPCR assay of gene expression**

The thermal cycling conditions comprised an initial denaturation step at 94°C for 5min, and 40 cycles at 94°C for 30sec, 60°C for 30sec and 72°C for 30sec. Transcripts of the TBP gene encoding the TATA-box binding protein (a component of the DNA- binding protein complex TFIID) were quantified as an endogenous RNA control. Quantitative values were obtained from the cycle number (Cq value), according to the manufacturer’s manuals.

**Detection of protein persulfidation**

Cells were lysed using HEN Buffer (0.5M EDTA, 20% SDS, 1% NP-40, 10mM Neocuproine and 0.1M HEPES, pH 7.4) supplemented with 5mM 4-chloro-7-nitrobenzofurazan (NBF-Cl, Sigma-Aldrich), as described (16). Proteins were then precipitated twice in the presence of methanol and chloroform at 14000rpm for 15min at 4°C, the pellets being resuspended in 50mM HEPES (pH 7.4) supplemented with 2% SDS. Once completely dissolved, protein concentration was determined by BCA assay and adjusted to 3mg/mL. The protein extracts prepared in presence of NBF-Cl were incubated for 30min at 37°C with 25μM click mix provided by Dr Filipovic (1mM Daz-2, 1mM Cy7.5 alkyne, 2mM TBTA Cu Complex, 4mM L- ascorbic acid, 30% acetonitrile and 20mM EDTA in PBS) and precipitated as described above. Proteins were subsequently resuspended in 50mM HEPES containing 2% SDS and separated using SDS-PAGE on 4-12% (w/v) polyacrylamide gels. Gels were fixed in 12.5% methanol and 4% acetic acid for 30min. The Cy7.5 signal corresponding to persulfidated proteins was recorded at 800nm and the NBF-adducts signal was recorded at 488nm on a Chemidoc Imager (BioRad). Persulfidation levels were assessed by normalizing the Cy7.5 intensity to Alexa488 (NBF-adducts) intensity.

**References of Supplemental Information**

1. Saurty-Seerunghen MS, Bellenger L, El-Habr EA, Delaunay V, Garnier D, Chneiweiss H, et al. Capture at the single cell level of metabolic modules distinguishing aggressive and indolent glioblastoma cells. Acta Neuropathol Commun. 2019;7:155.

2. Müller S, Cho A, Liu SJ, Lim DA, Diaz A. CONICS integrates scRNA-seq with DNA sequencing to map gene expression to tumor sub-clones. Bioinformatics. 2018;34:3217-9.

3. Gu Z, Eils R, Schlesner M. Complex heatmaps reveal patterns and correlations in multidimensional genomic data. Bioinformatics. 2016;32:2847-9.

4. Husson F, Josse J, Pagès J. Principal component methods - hierarchical clustering - partitional clustering: why would we need to choose for visualizing data? 2010 [Internet]. http://www.sthda.com/english/upload/hcpc_husson_josse.pdf

5. Gu Z, Gu L, Eils R, Schlesner M, Brors B. circlize Implements and enhances circular visualization in R. Bioinformatics. 2014;30:2811-2.

6. Manning C, Raghavan P, Schütze H. Introduction to Information Retrieval [Internet]. Cambridge University Press. 2008. https://nlp.stanford.edu/IR-book/information-retrieval-book.html

7. Soneson C, Robinson MD. Bias, robustness and scalability in single-cell differential expression analysis. Nat Methods. 2018;15:255-61.

8. Chen EY, Tan CM, Kou Y, Duan Q, Wang Z, Meirelles GV, et al. Enrichr: interactive and collaborative HTML5 gene list enrichment analysis tool. BMC Bioinformatics. 2013;14:128.

9. Kuleshov MV, Jones MR, Rouillard AD, Fernandez NF, Duan Q, Wang Z, et al. Enrichr: a comprehensive gene set enrichment analysis web server 2016 update. Nucleic Acids Res. 2016;44:W90-97.

10. Xie Z, Bailey A, Kuleshov MV, Clarke DJB, Evangelista JE, Jenkins SL, et al. Gene Set Knowledge Discovery with Enrichr. Curr Protoc. 2021;1:e90.

11. Ritchie ME, Phipson B, Wu D, Hu Y, Law CW, Shi W, et al. limma powers differential expression analyses for RNA-sequencing and microarray studies. Nucleic Acids Res. 2015;43:e47.

12. Chen H, Albergante L, Hsu JY, Lareau CA, Lo Bosco G, Guan J, et al. Single-cell trajectories reconstruction, exploration and mapping of omics data with STREAM. Nat Commun. 2019;10:1903.

13. Courte J, Renault R, Jan A, Viovy JL, Peyrin JM, Villard C. Reconstruction of directed neuronal networks in a microfluidic device with asymmetric microchannels. Methods Cell Biol. 2018;148:71-95.

14. Guyon J, Andrique L, Pujol N, Røsland GV, Recher G, Bikfalvi A, et al. A 3D Spheroid Model for Glioblastoma. J Vis Exp. 2020;(158).

15. Renault-Mihara F, Beuvon F, Iturrioz X, Canton B, De Bouard S, Léonard N, et al. Phosphoprotein enriched in astrocytes-15 kDa expression inhibits astrocyte migration by a protein kinase C delta-dependent mechanism. Mol Biol Cell. 2006;17:5141-52.

16. Zivanovic J, Kouroussis E, Kohl JB, Adhikari B, Bursac B, Schott-Roux S, et al. Selective Persulfide Detection Reveals Evolutionarily Conserved Antiaging Effects of S-Sulfhydration. Cell Metab. 2019;30:1152-1170.e13.

**Supplemental figure legends.**

**Figure S1, related to Methods. Identification of malignant and normal cells in N-10X dataset**

**A.** Cell clustering based on GMM-based CNV predictions. Heatmap representation of CNV (copy number variations) predictions (A1). Potential malignancy status assigned, following cell clustering based on CNV predictions, highlighted on UMAP representation (A2).

**B.** CNV predictions at canonical glioblastoma loci (Chr7 and 10). UMAP representation.

**C.** Expression of marker genes of normal cell types. Pan-immune cells (PTPRC), macrophages (ITGAM, FCGR3A, CD14), microglia (CSF1R, TMEM119), T-cells (CD2, CD3D) and oligodendrocytes (MOG, MAG). UMAP representation.

**D.** Cell clusters identified based on their repartition on UMAP plot. Hierarchical clustering followed by K-means clustering on UMAP components.

**E.** Cell malignancy status assigned based on CNV status prediction, marker gene expression and clustering results. UMAP representation.

**Figure S2, related to Methods. Identification of malignant and normal cells in PA-10X dataset**

**A.** Cell clustering based on GMM-based CNV predictions. Heatmap representation of CNV (copy number variations) predictions (A1). Potential malignancy status assigned, following cell clustering based on CNV predictions, highlighted on UMAP representation (A2).

**B.** CNV predictions at canonical glioblastoma loci (Chr7 and 10). UMAP representation.

**C.** Expression of marker genes of normal cell types. Pan-immune cells (PTPRC), macrophages (ITGAM, FCGR3A, CD14), microglia (CSF1R, TMEM119), T-cells (CD2, CD3D) and oligodendrocytes (MOG, MAG). UMAP representation.

**D.** Cell clusters identified based on their repartition on UMAP plot. Hierarchical clustering followed by K-means clustering on UMAP components.

**E.** Cell malignancy status assigned based on CNV status prediction, marker gene expression and clustering results. UMAP representation.

**Figure S3, related to Figure 1**

**A-B. Grouping strategy A**. Schematic representation of HCPC clustering strategy integrating UMAP components (comp). HCPC: Hierarchical clustering on principal components, PCA: Principal component analysis, PC: Principal components, UMAP: Uniform Manifold Approximation and Projection. **B**. Integrating UMAP components results in more homogeneous clusters, as shown by elimination of cell outliers on the UMAP representations (compare upper left and right panels) and the increase in the Silhouette width index (compare lower left and right panels). Clustering based on expression of the ten elements of the motility signature, and performed using 4916 glioblastoma cells from N-S dataset.

**C-F**. **Motility signature captures cells with similar profiles in independent datasets.**

Clustering malignant cells from D-S (**C**), N-10X (**D**) and PA-10X (**E**) datasets based on the motility signature genes. From left to right subpanels: UMAP representation; identification of cell groups with the highest and lowest mean motility scores (M^HIGH^, M^LOW^); Heatmap representation of the relative expression of each signature element per cluster; Contribution of each tumor to identified clusters. *: Clusters with mean motility score statistically different from each of the other clusters, p < 0.01, one-way ANOVA, Tukey's multiple comparisons test, NS: non-significant. **F**. Overlaps between lists of genes overexpressed (OEG) in M^HIGH^ compared to M^LOW^ cell groups from distinct datasets. High overlap between lists from datasets obtained with the same sequencing techniques, with 69.2% of OEG in M^HIGH^ cells from N-S dataset also overexpressed in M^HIGH^ cells from D-S dataset (4852/7010), and 86% of OEG in M^HIGH^ cells from N-10X dataset also overexpressed in M^HIGH^ cells from PA-10X dataset (6627/7707). When comparing N-S, D-S, N-10X and PA-10X to the 3 other datasets, lists of OEG in M^HIGH^ cells overlap by 56.6% (3590/6348), 53.2% (3590/6750), 48.4% (3590/7418) and 43.0% (3590/8348), respectively. Due to graphical constraints, two overlaps are not shown on the third venn diagram: 119 genes were identified as overexpressed in M^HIGH^ cells in the two SMART-seq2 datasets only, and 241 overexpressed in M^HIGH^ cells from N-S, D-S and N-10X datasets (not from PA-10X).

**Figure S4, related to Figure 1. Motility-related terms highlighted by ontology analysis of genes overexpressed in M^HIGH^ versus M^LOW^ cells from independent datasets**

Dot plot representation of enriched terms related to motility. Analyses performed using genes overexpressed with fold change >2 (**A**, D-S dataset, 1486 genes) and (**B**, N-10X dataset, 600 genes), and fold change >1.5 (**C**, PA-10X dataset, 338 genes). BH-adjusted p-value < 0.05. KEGG: Kyoto Encyclopedia of Genes and Genomes, BP: Biological processes, CC: Cellular components, MF: Molecular functions.

**Figure S5, related to Figure 1. Enrichment in EMT (A), oRG (B) and TEAD (C) gene modules previously associated with glioblastoma cell motility in M^HIGH^ cells.**

EMT: genes associated with epithelio-mesenchymal transformation and GB cell motility. oRG: genes signing for outer Radial Glia (oRG)-like malignant cell population with increased invasive behavior in GB. TEAD: TEAD1-regulated genes involved in GB cell motility.

Upper subpanels: Linear regression models between motility score and EMT, oRG and TEAD scores. p < 0.0001. Lower subpanels: Higher EMT, oRG and TEAD scores in M^HIGH^ versus M^LOW^ cells. *: p < 0.0001, Mann-Whitney test.

**Figure S6, related to Figures 3-5**

**A-C. Comparative migratory and invasive properties between glioblastoma PDC. A.** Cell migration on Matrigel assessed after 24h (5706**, R633 and P3). The microphotographs illustrate examples for each PDC. Scale bar = 200µm. Solid and dotted lines delineate the spheroid core and the migration area, respectively. The dot plot depicts the quantification of the migration. Mean ± SD, n = 6-12 independent biological samples, *: p < 0.05, Tukey's multiple comparisons test. **B.** Cell invasion into collagen assessed after 40h (5706**) and 23-25h (R633, P3). Microphotographs provide examples of a cell invasion assay for each PDC. Scale bars = 200µm. Solid and dotted lines delineate the spheroid core and the invasion area, respectively. The bar graph depicts the quantification of the invasion. Mean ± SD, n = 4-5 independent biological samples, *: p < 0.05, Tukey's multiple comparisons test. **C.** Quantification of cell invasion across Matrigel-coated transwells after 24 hours. 5706**, R633, and P3 PDC. Mean ± SD, n = 4-5 independent biological samples.

**D. Overlap of the MPST, MitoTracker and CellROX fluorescent signals in spheroids during migration on Matrigel.** Left upper panel: MPST-immunoreactive signal across cells used to generate spheroids. Right upper panel: 2.5D intensity plot of DAPI (blue) and MPST (orange) signals across and around spheroids after initiating migration on Matrigel. Individual peaks represent absolute signal intensities of each pixel. Lower panels: representative distribution profiles of the MPST, MitoTracker and CellROX signals across R633-spheroids. Signal analyses performed with the ZEN software (Zeiss), per manufacturer instructions.

**E-L. *MPST* knockdown. E.** Decreased *MPST* mRNA levels in PDC expressing sh*MPST-139.* Mean ± SD, n = 5-6 independent biological samples. *: p < 0.05, Mann-Whitney test. **F.** Decreased MPST protein levels in sh*MPST* expressing PDC. Western Blot analysis. 30 µg protein/lane. MPST MW: 33/35 kDa. *: p < 0.05, one-sample t-test. **G.** No major impact of *MPST* knockdown on cell viability. Cell viability assessed using Trypan Blue exclusion test. Mean ± SD, n = 5-12 independent biological samples, *: p < 0.05, Mann-Whitney test. 5706**, R633 and P3 PDC. **H.** Impact of *MPST* knockdown on energy metabolism. Left panel: representative Seahorse metabolic analysis of PDC expressing shCTL (red) or shMPST (blue). Oxygen consumption rates (OCR) were determined for each of the mitochondrial respiration steps after oligomycin, FCCP and rotenone/antimycin treatment. Middle and right panels: basal, maximal (max.), ATP-linked (ATP-linked resp., i.e. respiration sensitive to oligomycine reflecting the respiration used to drive mitochondrial ATP synthesis) and non-mitochondrial (non-mito resp.) respirations were derived from OCR measurements. Mean ± SD, n=3 independent biological samples, *: p < 0.05, unpaired t-test with Welch’s correction. 5706** and R633 PDC. **I.** Decreased *MPST* mRNA levels in shMPST142-PDC compared to shControl-PDC. Q-PCR assay. Mean ± SD, n = 2-3 independent biological samples. **J.** No change in cell viability upon shMPST-142 expression assessed using Trypan Blue exclusion test. Mean ± SD, n = 3-5 independent biological samples, unpaired t-test with Welch correction. **K.** Decreased cell migration upon *MPST* knockdown. Cell migration assessed on Matrigel 7h post-seeding. Mean ± SD, n = 8-9 independent biological samples, *: p < 0.05, Mann-Whitney test. Quantification of cell invasion across Matrigel-coated transwells after 24 hours. 5706**, R633, and P3 PDC. Mean ± SD, n = 3-5 independent biological samples. **L. *MPST* knockdown decreases tumor burden until experiment end-points.** DPG: days post-graft. Mean ± SD, *: p < 0.01, Mann-Whitney test. 5706**-PDC: 86 DPG, n = 6 mice per group; 148 DPG, n = 5 shCTL and n = 6 shMPST. P3-PDC: 21 DPG, n = 6 mice per group; 67 DPG, n = 6 mice per group.

**Figure S7, related to Figures 4b**

Full-size uncropped original image of the Western blot shown in the inset above graph in Figure 4b illustrating decreased MPST protein levels in sh*MPST*-PDC (Western Blot analysis, MW: 33/35 kDa).

**Figure S8, related to Figures 5b-c**

In-gel detection of persulfidation levels. Dimedone switch method with Cy7.5 as a P-SSH reporting molecule. Full-size uncropped original images of the gels used to illustrate results depicted in Figure 5b and 5c. Graphs illustrate the P-SSH levels calculated as a ratio of Cy7.5 (800nm)/NBF-protein adducts signal (488nm) within low (<28kDa), high (>28kDa) and all MW ranges. Mean±SD, n=3 independent biological samples, *p<0.05, unpaired t-test.

**Supplemental table legends.**

**Table S1. Motility signature genes**

Sheet 1: description of the contents

Sheet2: list of genes in the motility signature.

Sheet3: Pearson correlation analysis between the motility signature genes in the N-S dataset.

**Table S2. Differentially expressed genes between M^HIGH^ and M^LOW^ cells (sheets 2-6) and oRG, EMT and TEAD-induced migration signatures (Sheet 7).**

Sheet 1: description of the contents

Sheet2: differentially expressed genes between M^HIGH^ and M^LOW^ cells, N-S dataset

Sheet3: differentially expressed genes between M^HIGH^ and M^LOW^ cells, D-S dataset

Sheet4: differentially expressed genes between M^HIGH^ and M^LOW^ cells, N-10X dataset

Sheet5: differentially expressed genes between M^HIGH^ and M^LOW^ cells, PA-10X dataset

Sheet6: genes overexpressed in M^HIGH^ versus M^LOW^ cells in the 4 datasets

Sheet7: oRG, EMT and TEAD-induced migration signatures

**Table S3. Results of gene ontology (GO) and KEGG pathway analyses performed with the list of genes overexpressed in M^HIGH^ versus M^LOW^ cells**

Sheet 1: description of the contents

Sheet 2: results obtained using genes overexpressed in M^HIGH^ versus M^LOW^ cells with FC >2 (N-S dataset)

Sheet 3: results obtained using genes overexpressed in M^HIGH^ versus M^LOW^ cells with FC >2 (D-S dataset)

Sheet 4: results obtained using genes overexpressed in M^HIGH^ versus M^LOW^ cells with FC >2 (N-10X dataset)

Sheet 5: results obtained using genes overexpressed in M^HIGH^ versus M^LOW^ cells with FC >1.5 (PA-10X dataset)

**Table S4. KEGG pathway analysis of metabolism genes overexpressed in glioblastoma cells with high motile potential**

Sheet1: Description of the contents

Sheet2: List of metabolism genes upregulated in M^HIGH^ versus M^LOW^ cells from each dataset

Sheet3: Results of the KEGG pathway analysis performed on metabolism genes upregulated in M^HIGH^ versus M^LOW^ cells from N-S dataset (BH-adjusted p-value <0.05)

Sheet4: Results of the KEGG pathway analysis performed on metabolism genes upregulated in M^HIGH^ versus M^LOW^ cells from D-S dataset (BH-adjusted p-value <0.05)

Sheet5: Results of the KEGG pathway analysis performed on metabolism genes upregulated in M^HIGH^ versus M^LOW^ cells from N-10X dataset (BH-adjusted p-value <0.05)

Sheet6: Results of the KEGG pathway analysis performed on metabolism genes upregulated in M^HIGH^ versus M^LOW^ cells from PA-10X dataset (BH-adjusted p-value <0.05)

**Table S5. Metabolism genes overexpressed in S3-S1 branch compared to each other branch**

**Table S6. List of resources, materials, bio-informatics tools, corresponding websites and references.**

**Supplemental datafile legends.**

**Datafiles S1 and S2**. Scripts used in this study
