# Supplementary material for: Glioblastoma cell motility depends on enhanced oxidative stress coupled with mobilization of a sulfurtransferase
Source: Cell Death Dis. 2022 Oct 30;13(10):913. doi: 10.1038/s41419-022-05358-8 (PMC9618559; doi:10.1038/s41419-022-05358-8)
Supplement: Supplementary file 2 — Supplemental Figures [file 41419_2022_5358_MOESM2_ESM.pdf]

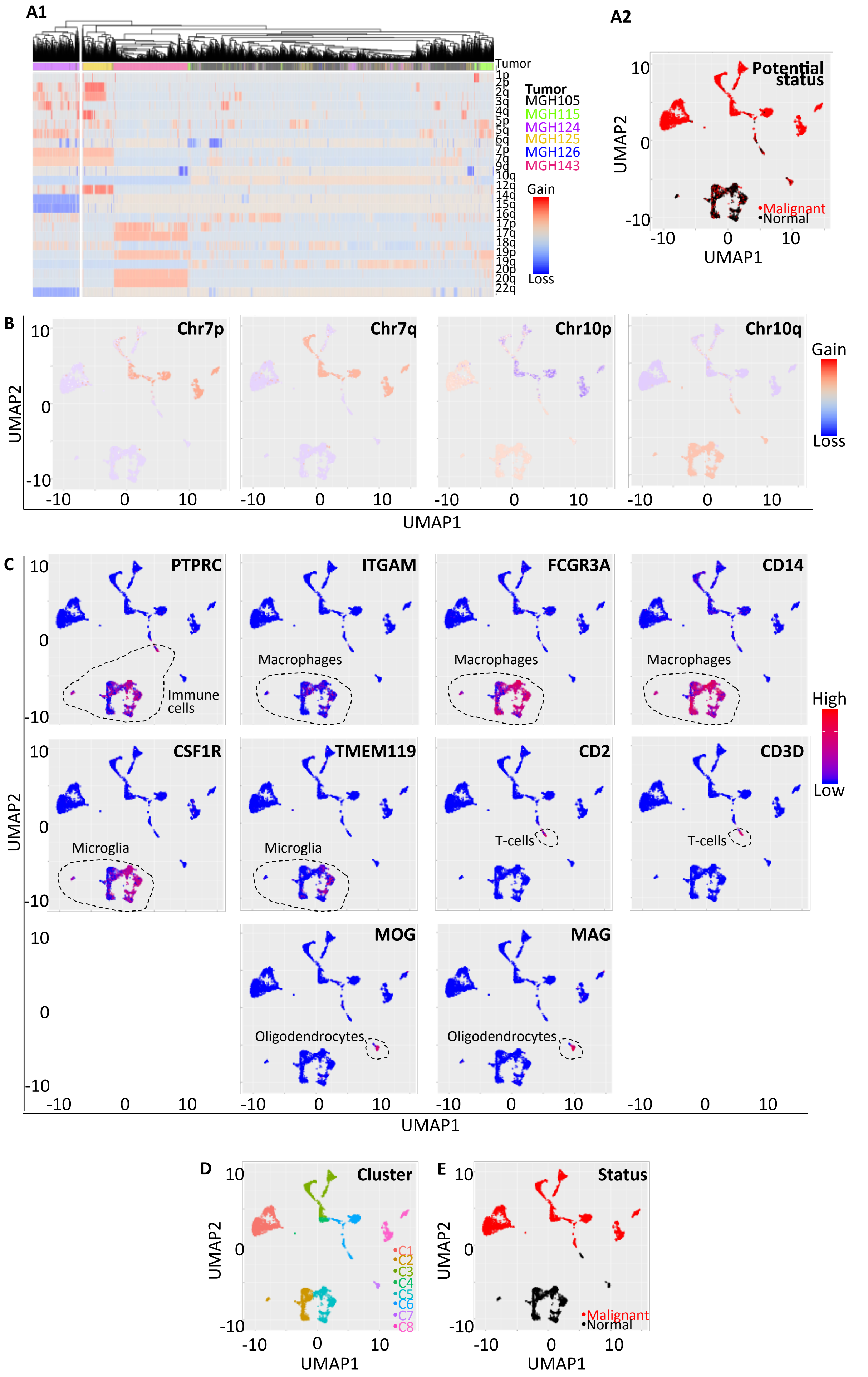

Supplementary Figure S1, related to Methods. Identification of malignant and normal cells in N-10X dataset.

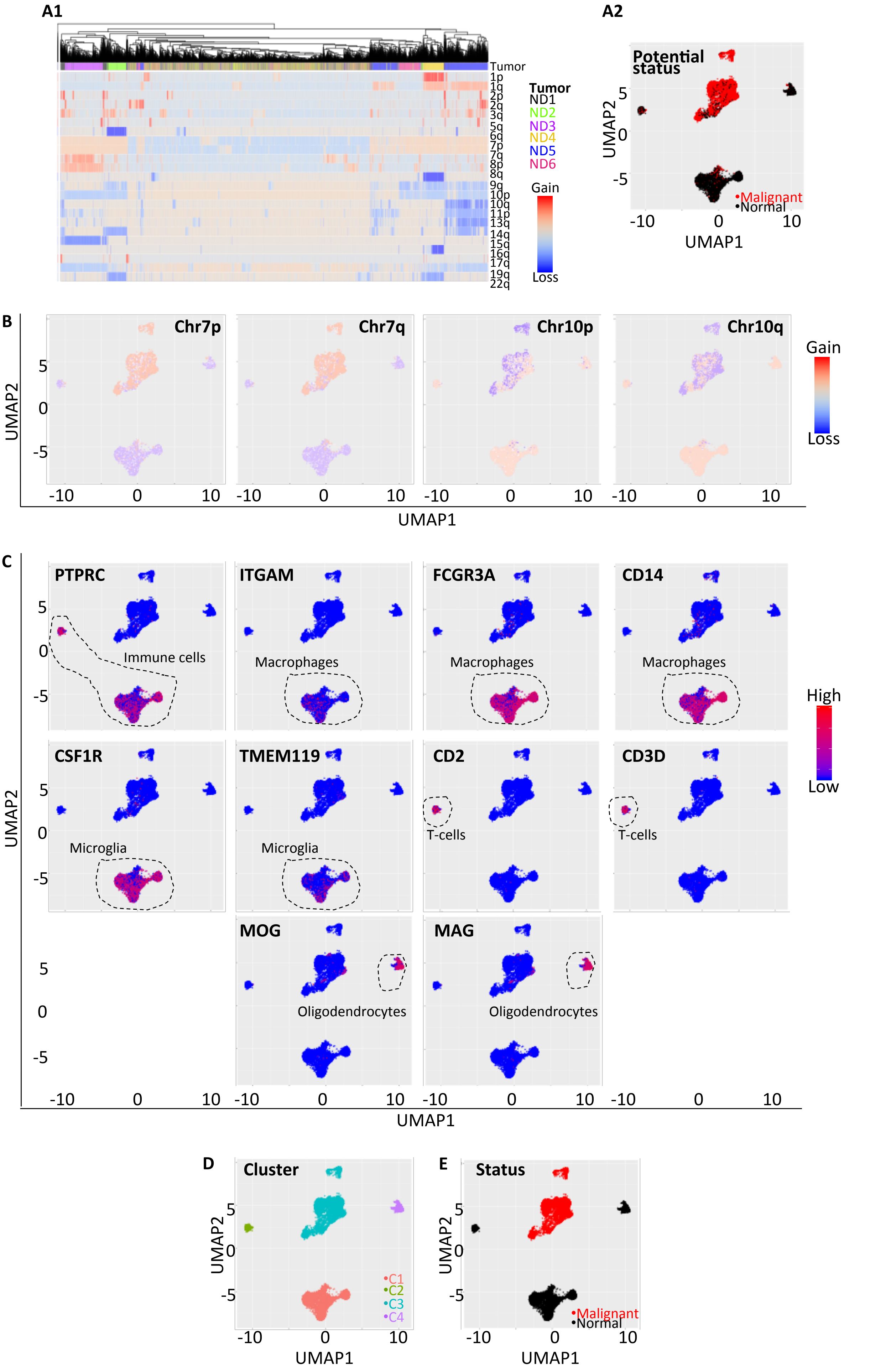

Supplementary Figure S2, related to Methods. Identification of malignant and normal cells in PA-10X dataset.

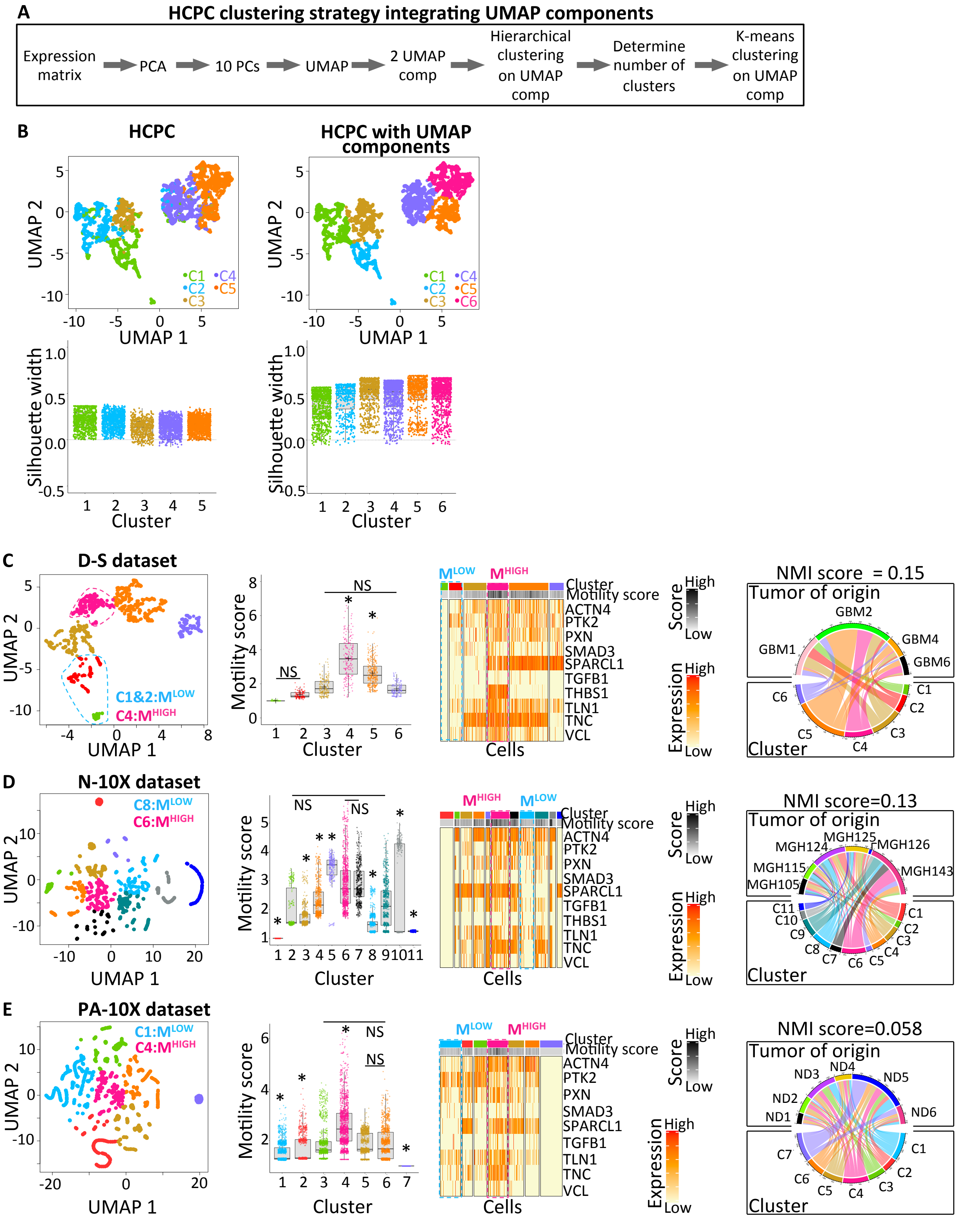

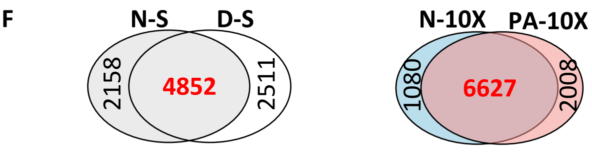

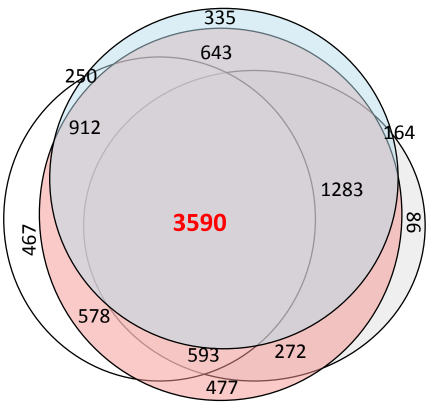

Supplementary Figure S3, related to Figure 1.
A-B. Grouping strategy.
C-F. Motility signature captures cells with similar profiles in independent datasets.

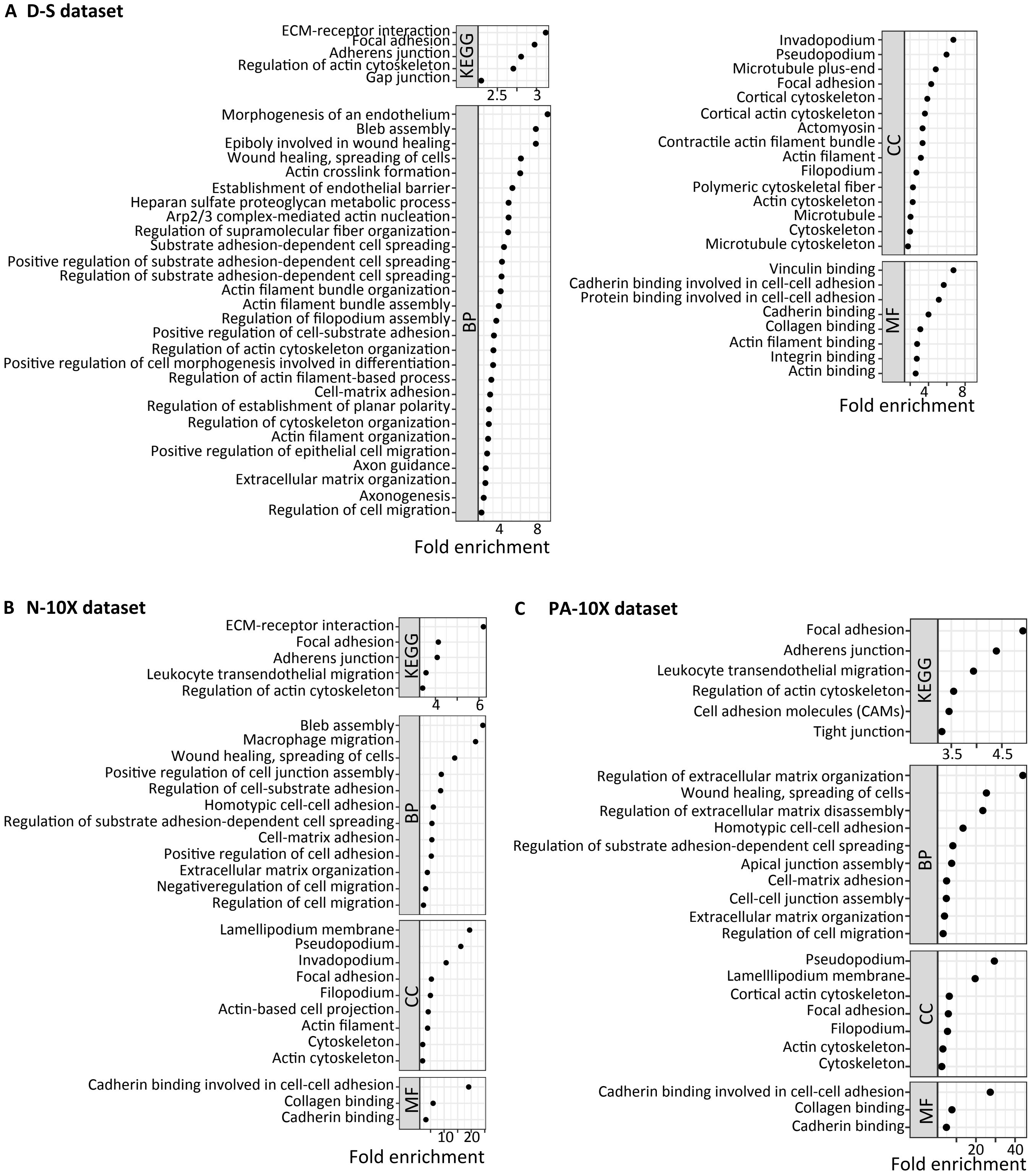

Supplementary Figure S4, related to Figure 1. Motility-related terms highlighted by ontology analysis of genes overexpressed in M^HIGH versus M^LOW cells from independent datasets.

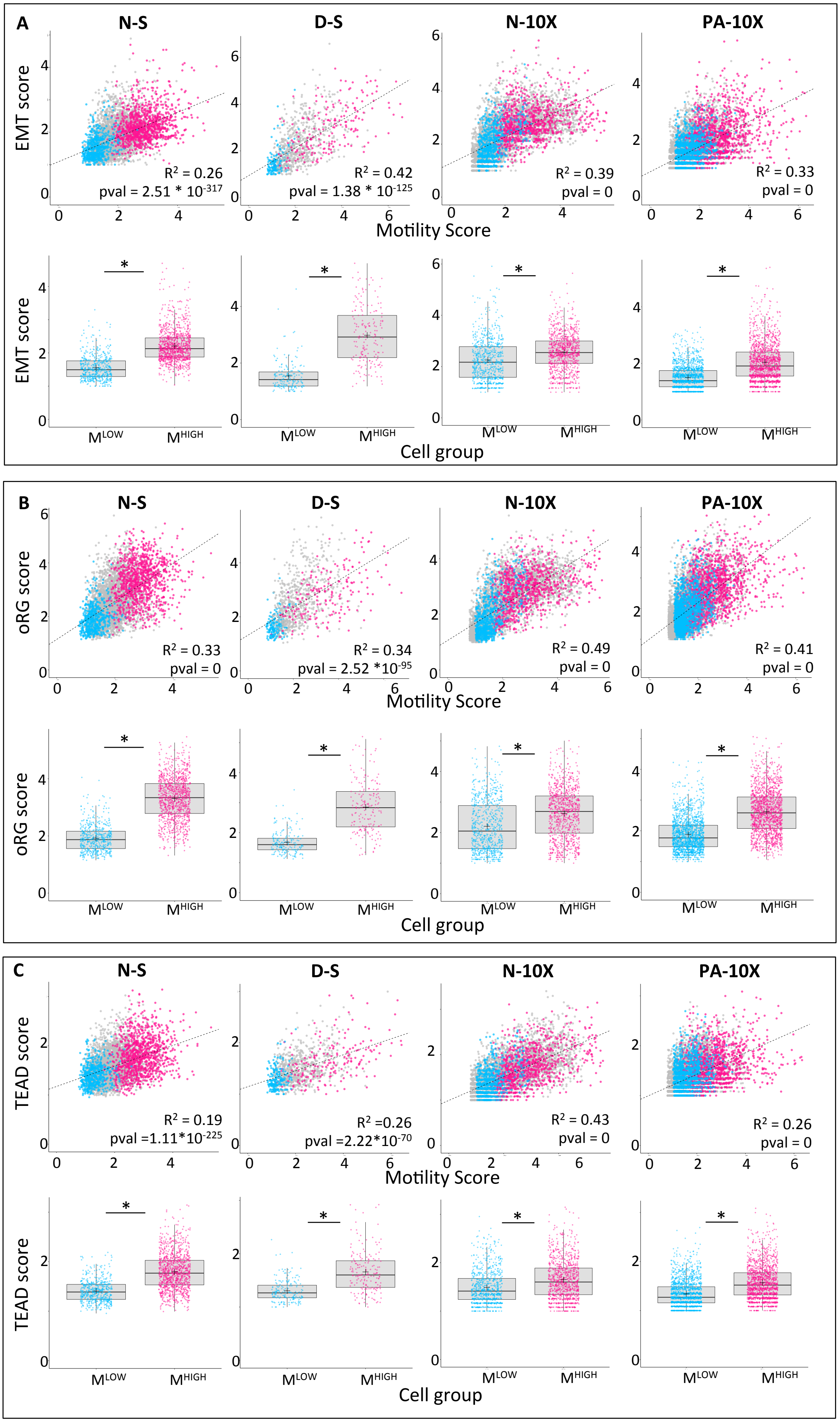

Supplementary Figure S5 , related to Figure 1. Enrichment in EMT (A), oRG (B) and TEAD (C) gene modules previously associated with glioblastoma cell motility in M^{HIGH} cells.

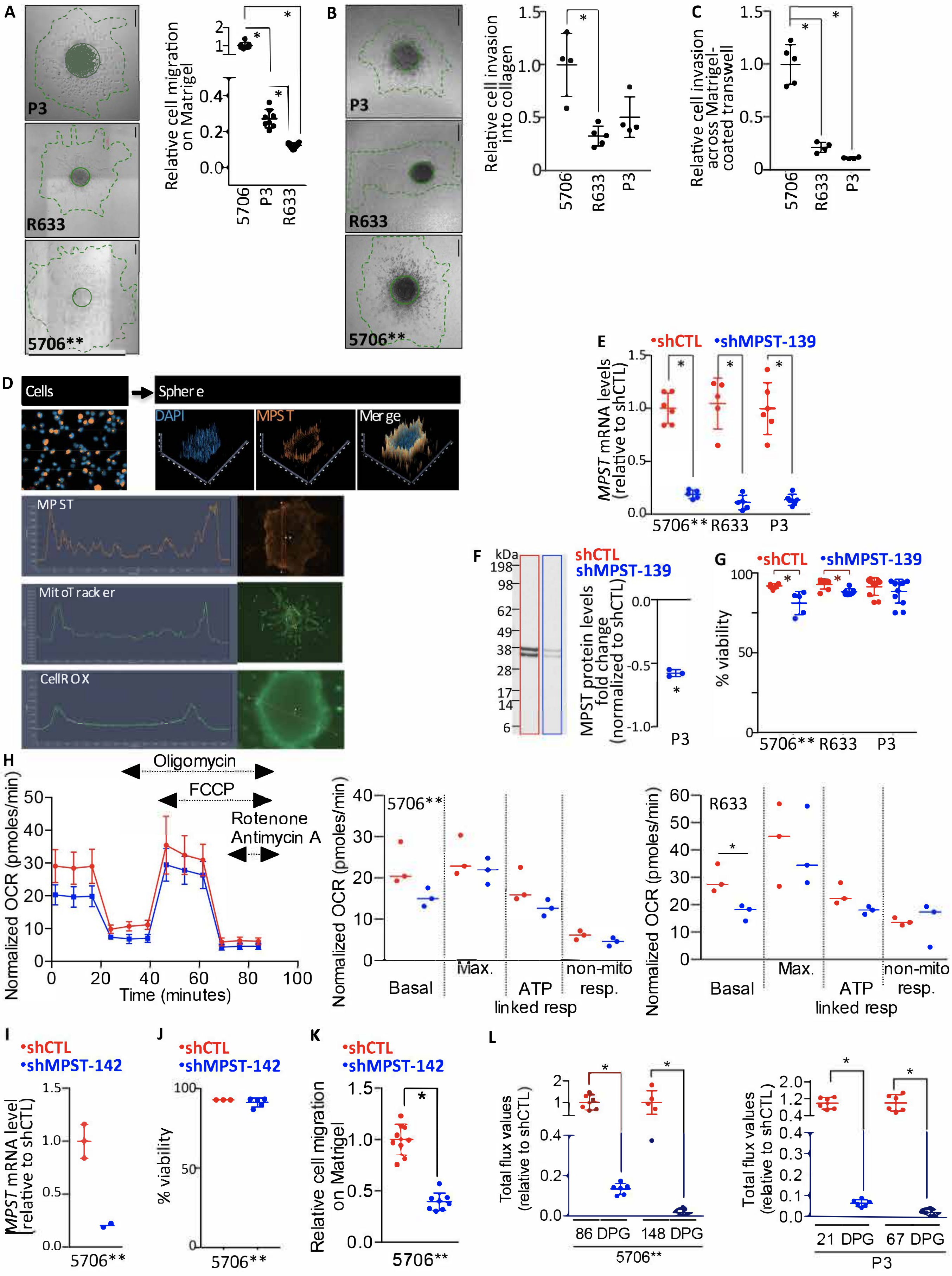

Supplementary Figure 6, related to Figures 3-5.

A-C. Comparative migratory and invasive properties between glioblastoma PDC.

D. Overlap of the MPST, MitoTracker and CellROX fluorescent signals in cellular spheres during migration-on-Matrigel.

E-G and I-K. *MPST* knockdown.

H. Impact of MPST knockdown on energy metabolism.

L. *MPST* knockdown decreases tumor burden until experiment end-points.

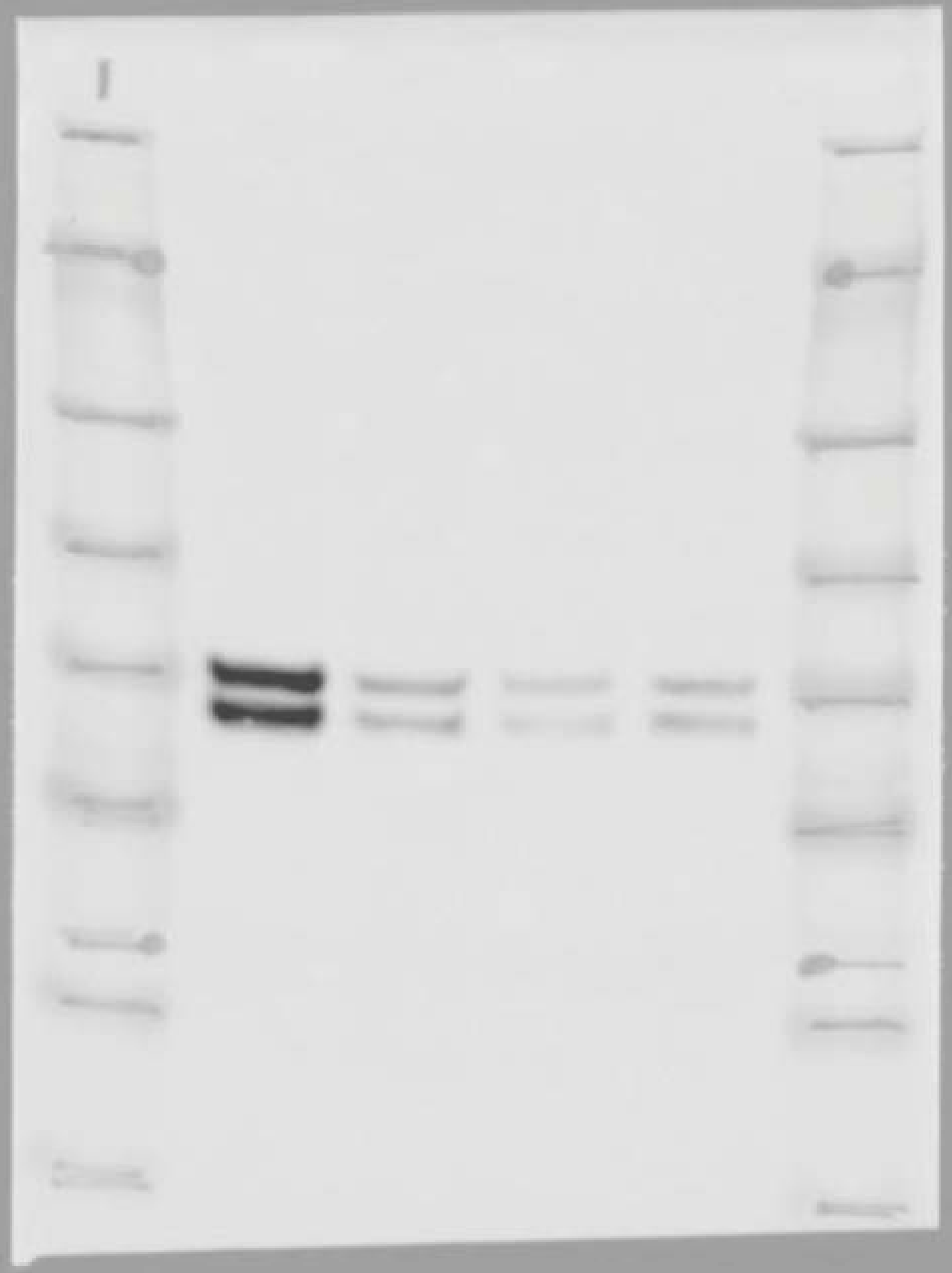

Supplementary Figure 7, related to Figure 4b.
Full-size uncropped original image of the Western blot shown in the inset above graph in Figure 4b illustrating decreased MPST protein levels in shMPST-PDC (Western Blot analysis, MW: 33/35 kDa).

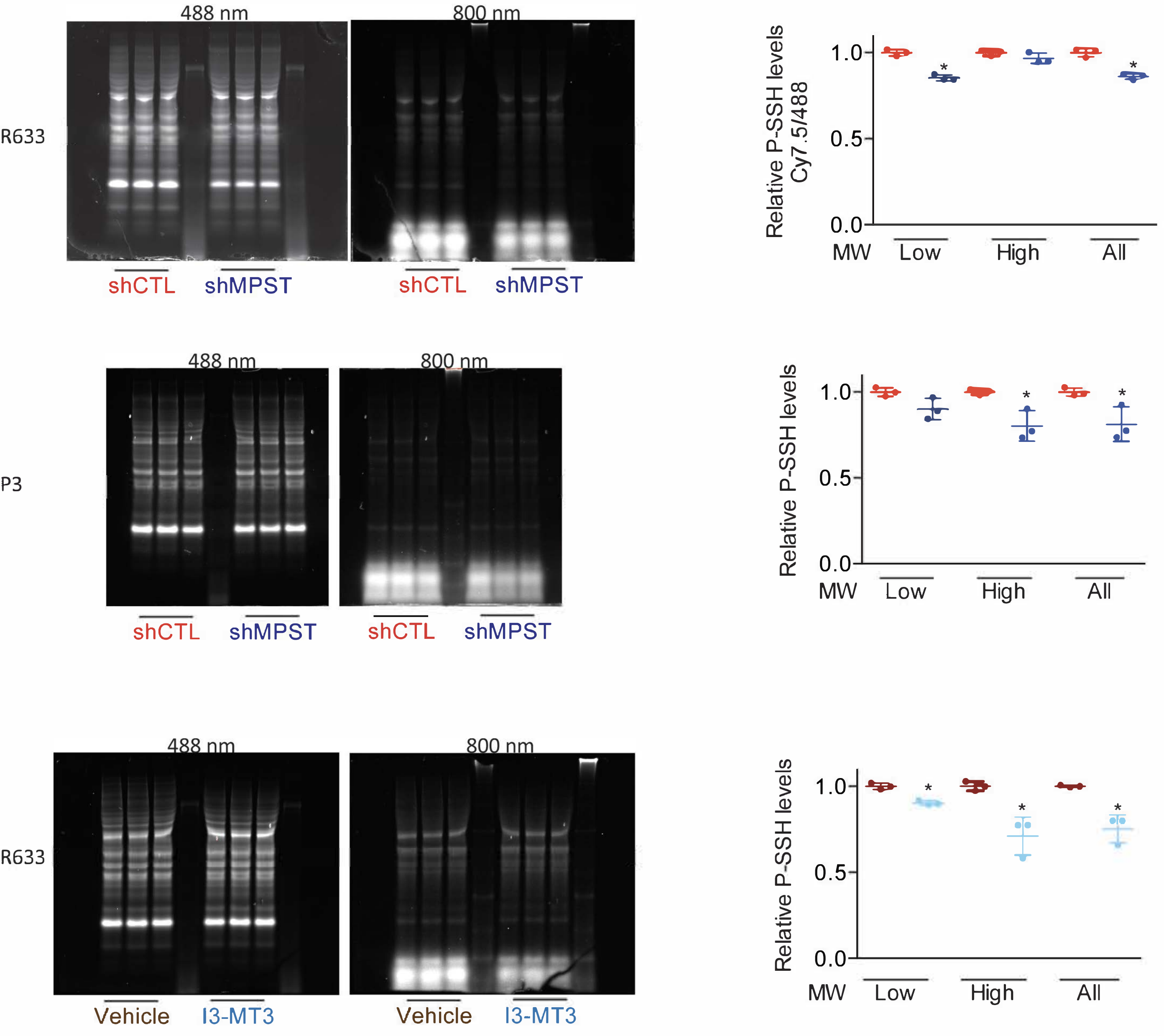

Supplementary Figure 8, related to Figures 5b-c.
